# Quantitative Proteomic Analysis of the Rice (*Oryza sativa* L.) Salt Response

**DOI:** 10.1371/journal.pone.0120978

**Published:** 2015-03-20

**Authors:** Jianwen Xu, Hongxia Lan, Huimin Fang, Xi Huang, Hongsheng Zhang, Ji Huang

**Affiliations:** 1 State key laboratory of crop genetics and germplasm enhancement, Nanjing Agricultural University, Nanjing, 210095, China; 2 Jiangsu Collaborative Innovation Center for Modern Crop Production, Nanjing, 210095, China; 3 College of Life Sciences, Nanjing Agricultural University, Nanjing, 210095, China; CSIR-National Botanical Research Institute, INDIA

## Abstract

Salt stress is one of most serious limiting factors for crop growth and production. An isobaric Tags for Relative and Absolute Quantitation (iTRAQ) approach was used to analyze proteomic changes in rice shoots under salt stress in this study. A total of 56 proteins were significantly altered and 16 of them were enriched in the pathways of photosynthesis, antioxidant and oxidative phosphorylation. Among these 16 proteins, peroxiredoxin Q and photosystem I subunit D were up-regulated, while thioredoxin M-like, thioredoxin x, thioredoxin peroxidase, glutathione S-transferase F3, PSI subunit H, light-harvesting antenna complex I subunits, chloroplast chaperonin, vacuolar ATP synthase subunit H, and ATP synthase delta chain were down-regulated. Moreover, physiological data including total antioxidant capacity, peroxiredoxin activity, chlorophyll a/b content, glutathione S-transferase activity, reduced glutathione content and ATPase activity were consistent with changes in the levels of these proteins. The levels of the mRNAs encoding these proteins were also analyzed by real-time quantitative reverse transcription PCR, and approximately 86% of the results were consistent with the iTRAQ data. Importantly, our data suggest the important role of PSI in balancing energy supply and ROS generation under salt stress. This study provides information for an improved understanding of the function of photosynthesis and PSI in the salt-stress response of rice.

## Introduction

Salt stress is a major obstacle limiting plant growth and development [1]. Under high-salinity conditions, photosynthesis, protein synthesis and metabolism are significantly affected. The mechanism of the salt stress response has been widely studied over the past several decades [2]. To cope with salinity conditions, plants have evolved many biochemical and molecular mechanisms that likely act both additively and synergistically [3].

Salt stress can induce reactive oxygen species (ROS) generation and cause oxidative damage to the cell and metabolic processes [4]. Antioxidants such as glutathione reductase (GR), superoxide dismutase (SOD), catalase (CAT), ascorbate peroxidase (APX) and peroxiredoxin (POD) can provide oxidative stress resistance [5]. Photosynthesis is a well-established ROS source in plants, as ROS such as hydrogen peroxide are generated during photosynthesis [4, 6]. It was reported that ROS are primarily generated in chloroplasts under several types of stress [7]. In plants, NADPH oxidase can transfer electrons from the NADPH generated by photosystem I (PSI) to an electron acceptor, leading to the formation of ROS [8]. Salt stress can decrease chlorophyll contents [9] and suppress photosynthesis [10]; however, increased photosynthesis has been reported under low salt concentrations [11]. Photosystem II (PSII) activities gradually decrease with increasing salt concentration [12]. Salt stress itself has been shown to have no direct effects on PSII activity other than blockage of electron transport between QA and QB (the PSII primary and secondary quinine electron acceptors) [13, 14]. Interestingly, PSI activity is increased in *Cyanobacteria* under salt stress [15], while in rice, it gradually decreases with increasing salt concentration [12]. Increased PSI activity in salt-adapted *Cyanobacteria* cells was shown to potentially protect PSII from excessive excitation energy under salt stress [16]. Differences between the two species may arise from the different response mechanisms developed during their evolution.

During photosynthesis, the majority of electrons flow linearly from water through PSII, the cytochrome *b6/f* complex and PSI to NADP^+^, powers the production of NADPH and ATP. Additionally, there is a cyclic electron flow around PSI [6] that provides a mechanism whereby ATP production can be increased relative to NADPH production. In the linear mode, electrons generated on the stromal side of PSI are transferred to NADP^+^ via Fd and Fd-NADP^+^ reductase (FNR). In the cyclic mode, Fd is reoxidized on the stromal side of the cytochrome *b6/f* complex and the electrons are back-transferred to P700, which is maintained in a reduced state [17]. The cyclic electron flow around PSI was shown to be enhanced during salt stress [16].

There have been many proteomic studies of rice under salt treatment. Most research has been carried out using traditional 2-dimensional electrophoresis (2-DE). Kim *et al*. used 2-DE to identify 33 significantly regulated proteins in salt-stressed rice leaves [18]. Using the same method, Parker *et al*. identified 11 salt stress response proteins in rice leaf lamina [19]. Yan *et al*. identified 10 differentially expressed proteins in salt-stressed rice roots [20], and Cheng *et al*. identified 18 salt stress response proteins [21]. Recently, an isobaric Tags for Relative and Absolute Quantitation (iTRAQ) approach identified at least 521 proteins that responded to salt stress in suspension-cultured rice cells [22]. To date, there have been no reports investigating the rice shoot proteome under conditions of salt stress using quantitative proteomic methods.

In this study, an iTRAQ-based quantitative proteomics approach was used to identify proteins in rice shoots that responded to salt stress. The differentially expressed proteins are primarily involved in the photosynthetic, antioxidant and oxidative phosphorylation pathways. Using the iTRAQ data and our physiological and biochemical analyses, we propose a working model for proteome reprogramming in rice under salt stress. These results provide information to increase understanding of the function of photosynthesis and PSI in the salt stress response.

## Material and Methods

### Plant materials and growth

*Japonica* rice cultivar Zhonghua11 (ZH11) plants were grown in the plastic plates (40 cm×25 cm×5 cm) contained nutritive soil (Fanghua Horticulture Company, Nanjing, Jiangsu, China) in a growth chamber with a 16 h photoperiod at 28°C/26°C (day/night). At the four-leaf stage, plates were transferred to a solution containing 150 mM NaCl and controls were transferred into distilled water. After twenty-four hours of treatment, the shoots of 30 seedlings from each group were combined for the protein extraction. Seedlings treated with either 150 mM NaCl or distilled water (control) were used for real-time quantitative reverse transcription PCR (qRT-PCR) and physiological assays.

### Protein extraction, iTRAQ labeling and strong cation exchange (SCX) fractionation

The proteins were isolated using trichloroacetic acid (TCA) [23]. About 20 mg of pooled protein from control or salt stress treated samples was labeled with iTRAQ reagents according to the manufacturer’s instructions (iTRAQ Reagents Multiplex kit, Applied Biosystems/MDS Sciex, Foster City, CA, USA). Control sample was labeled with 113 iTRAQ reagents, and salt stress treated sample labeled with 116. Reactions were quenched with glycine (10 mM). The labeled samples were pooled, vacuum-dried and subjected to SCX fractionation. Eight fractions were collected and used for LC-MS/MS analysis. SCX fractionation and LC-MS/MS analysis were performed as previously described [24].

### Protein identification and quantification

The MS and MS/MS data were searched against the NCBI rice Nipponbare database containing 213,855 proteins using Mascot 2.3.02. The peptide and protein data were extracted using high peptide confidence and top one peptide rank filters. The false discovery rate (FDR) was calculated by peptide sequence analysis with a decoy database. High confidence peptide identifications were obtained by setting a target FDR threshold of 1% at the peptide level. Relative quantitation of proteins was performed based on the relative intensities of reporter ions released during the MS/MS peptide fragmentation. Only unique peptides for each identified protein were used to determine the relative protein contents of the samples.

### Bioinformatics analysis

The annotation of the identified proteins were carried out based on the molecular functions, cellular components and biological processes listed in the Gene Ontology (GO) database (http://www.geneontology.org) in compliance with GO standards. Pathway grouping was performed using the Kyoto Encyclopedia of Genes and Genomes (KEGG) database (http://www.genome.jp/kegg/). The MapMan tool [25] was used to analyze the metabolic and signaling changes in the iTRAQ data. Unlike KEGG pathway mapping, MapMan considers the expression value of each differentially expressed protein. The pathways were produced by loading the differentially expressed proteins and Log2-transformed expression values into a locally installed MapMan program and were displayed by color intensity.

### Real-time quantitative reverse transcription PCR

Total RNA was extracted from four-leaf stage seedling shoots using Trizol reagent (Invitrogen, Foster City, CA, USA), and cDNA was synthesized using Super Script III reverse transcriptase (Invitrogen, Foster City, CA, USA) according to the manufacturer’s instructions. Real-time quantitative reverse transcription PCR (qRT-PCR) was performed using the cDNA template and gene-specific primer pairs for several stress-related mRNAs (listed in S1 Table).

### Physiological assays

Physiological parameters were analyzed by using the commercial kits (Jiancheng Bioengineering Institute, Nanjing, Jiangsu, China) for assaying total antioxidant capacity (T-AOC), reduced glutathione (GSH), peroxiredoxin (POD), glutathione S-transferase (GST) and ATPase activities according to the manufacturer’s instructions.

## Results

### Protein identification

**Fig 1:**
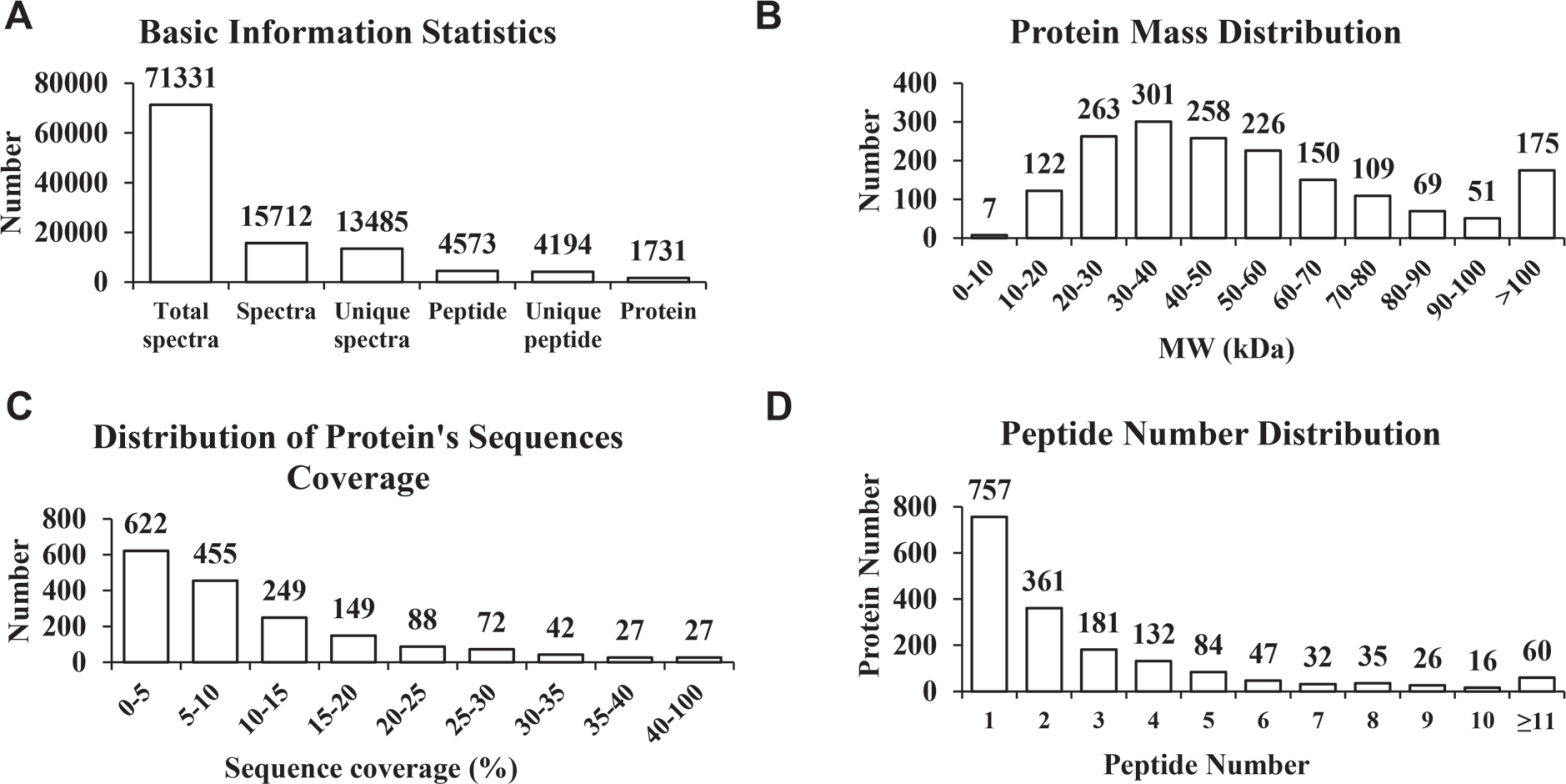
Generation of iTRAQ data. *(A)* Mascot identification statistics. “Total Spectra” indicates the number of all the spectra spectrograms detected by iTRAQ. “Spectra” indicates the number of identified spectra spectrograms. “Unique Spectra” indicates the number of spectra spectrograms matching the unique peptide sequences. “Peptide” indicates the number of identified peptide sequences. “Unique Peptide” indicates the number of unique peptide sequences in the identified proteins. “Protein” indicates the number of identified proteins. *(B)* The mass distribution of the identified proteins. The numbers of proteins distributed in different rang of molecular mass were shown in the graph. *(C)* The peptide sequence coverage for the identified proteins. The numbers of proteins distributed in different rang of peptide sequence coverage were shown in the graph. *(D)* The distribution of the number of peptides for the identified proteins. Based on the number of matched peptides, the identified proteins were grouped. The numbers of proteins in each group were counted in the graph.

Rice seedling shoots subjected to normal or salt stress conditions for 24 h were collected and used for iTRAQ analysis. A total of 71,331 spectra were detected by iTRAQ and 15,172 were identified. Among the identified spectra, 13,485 matched 4,573 peptides, with 4,194 unique ones. In total, 1,731 proteins were identified (Fig. 1A). Among these proteins, 1,048 were between 20 to 60 kDa, 129 between 0 to 20 kDa, 379 between 60 to 100 kDa, and 175 over 100 kDa (Fig. 1B). The peptide sequence coverage was primarily less than 20% (Fig. 1C). 757 proteins had one identified peptide, 361 had two, 60 had more than 11, and the left had 3 to 10 (Fig. 1D).

**Fig 2:**
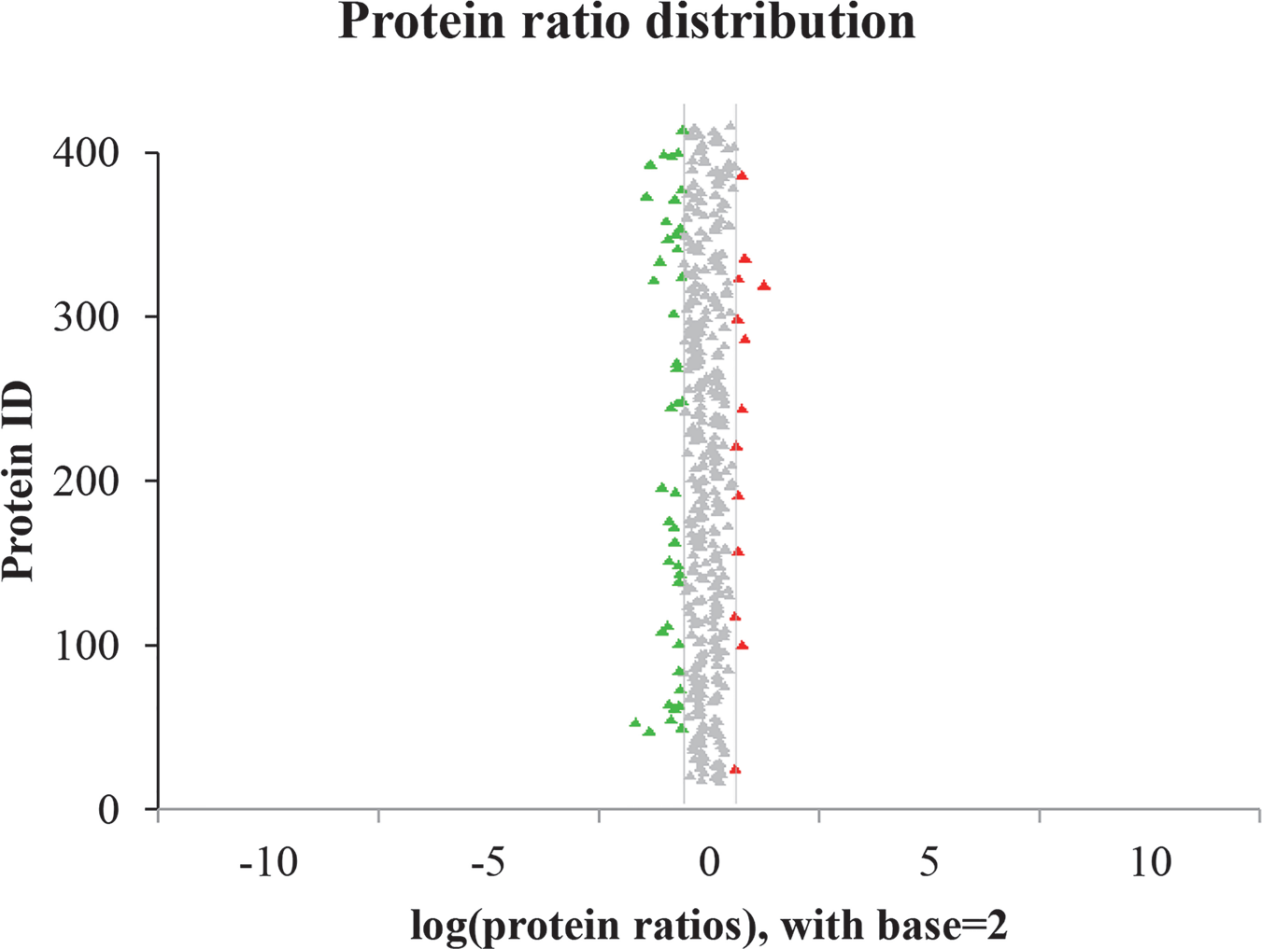
Ratio distributions for the identified proteins. The changes in rice protein contents in response to salt stress were analyzed. The horizontal axis displays the base Log2-transformed ratios. The red point indicates that the ratio was greater than 1.5, and the green point indicates that the ratio was less than 0.67.

The proteins from rice shoot with or without salt stress treatment were quantitatively analyzed. If the fold change was greater than 1.5 or less than 0.67 and the *p*-value was less than 0.05, the protein was defined as being differentially expressed. Among the 56 differentially expressed proteins, 43 were up-regulated and 13 were down-regulated (Fig. 2, Table 1). These proteins were further identified by bioinformatics analysis.

**Table 1:** The differentially expressed proteins in rice under salt stress.

Pathway	Accession	Description	Cov^a^	Spectrum^b^	Unique Spectrum^c^	Peptide^d^	Unique Peptide^e^	Fold ratio^f^
Photosynthesis	gi|29367391	chloroplast photosystem I reaction center subunit II precursor-like protein (PsaD)	14.8	31	31	2	2	1.585
	gi|3885894	photosystem-1 H subunit GOS5 (PsaH)	7.7	4	4	1	1	0.624
	gi|3789954	chlorophyll a/b-binding protein precursor (Lhca1)	5.4	5	5	1	1	0.581
	gi|34393511	putative photosystem I antenna protein (Lhca2)	16.3	4	4	2	2	0.635
	gi|3789952	chlorophyll a/b-binding protein precursor (Lhca4)	20.1	12	12	3	3	0.549
	gi|18855008	putative chloroplast chaperonin	20.7	3	3	2	2	0.651
Antioxidant activity	gi|51090743	putative peroxiredoxin Q	19.8	27	27	5	5	1.688
	gi|46389828	putative thioredoxin peroxidase	32.9	14	11	5	4	0.66
	gi|57899183	thioredoxin M-like	10.6	2	2	1	1	0.608
	gi|32487506	thioredoxin x	6.1	4	4	1	1	0.477
	gi|11177845	putative glutathione S-transferase OsGSTF3	16.1	8	8	3	3	0.635
Oxidative phosphorylation	gi|3885882	inorganic pyrophosphatase	8.4	3	3	1	1	0.623
	gi|28564802	putative vacuolar ATP synthase subunit H	1.5	3	3	1	1	0.617
	gi|51535416	putative ATP synthase delta chain	24.9	6	6	4	4	0.649
	gi|41052565	putative ATP synthase	16.2	3	3	3	3	0.48
	gi|108864431	Acyl carrier protein 2, chloroplast precursor	6.8	3	3	1	1	0.419
Transcription factor	gi|77553225	Carboxyvinyl-carboxyphosphonate phosphorylmutase, putative, expressed	5.9	6	6	2	2	0.648
	gi|24431603	Putative transcription factor	4.9	5	4	2	1	1.598
	gi|55296302	putative MAR binding filament-like protein 1	8	11	11	4	4	1.684
IAA	gi|77552436	auxin-repressed protein-like protein ARP1, putative, expressed	9.6	2	2	1	1	2.382
Jasmonate	gi|33358444	hydroperoxide lyase	3.3	5	5	1	1	0.491
Ethylene /Jasmonate	gi|34015153	putative CBS domain containing protein	13.7	3	3	2	2	0.662
Calcium regulation	gi|28209481	expressed protein	13.9	4	4	2	2	0.532
G-proteins	gi|5922611	putative small GTP-binding protein Bsar1a	6.7	4	4	1	1	0.579
Carbon & Nutrients	gi|50878396	putative P-II nitrogen sensing protein	5.2	4	4	1	1	0.632
RNA synthesis	gi|41052905	putative small nuclear ribonucleoprotein polypeptide D3	6.8	4	4	1	1	0.608
	gi|113578236	Os05g0154800	3.2	2	2	1	1	0.625
Protein synthesis	gi|50725625	putative acidic ribosomal protein P1a	10	4	4	1	1	0.372
	gi|25553579	putative ribosomal protein S18	21.1	9	9	3	3	0.57
	gi|50252685	putative ribosomal protein L10a	5.1	2	2	1	1	0.604
	gi|11974	ribosomal protein S2	10.6	3	3	2	2	0.614
	gi|37805854	putative ribosomal protein L34	6.8	4	4	1	1	1.563
Protein degradation	gi|14495192	putative 26S proteasome subunit RPN9b	3.1	2	2	1	1	0.519
	gi|11094192	26S proteasome regulatory particle triple-A ATPase subunit4	6.8	7	4	2	1	0.622
Nucleosome	gi|12039318	histone H4	42.7	14	14	4	4	0.314
	gi|6319146	H2A protein	7.2	3	3	1	1	0.396
	gi|3885890	histone H3	5.1	4	4	1	1	0.533
Regulation of actin cytoskeleton	gi|29124123	putative actin depolymerizing factor	18	5	5	2	2	0.576
	gi|34851127	actin	35.8	49	2	11	1	1.758
Biosynthesis of secondary metabolites	gi|27260946	putative isopentenyl pyrophosphate:dimethyllallyl pyrophosphate isomerase	11.3	6	6	2	2	0.508
Glyoxylate and dicarboxylate metabolism	gi|34393921	putative isocitrate lyase	4.7	2	2	2	2	1.679
Phenylpropanoid biosynthesis	gi|5257275	putative caffeoyl-CoA O-methyltransferase 1	4	2	2	1	1	0.588
Porphyrin and chlorophyll metabolism	gi|21686526	ferritin	12	4	4	2	2	0.391
None	gi|125600465	hypothetical protein OsJ_24479	7.6	5	5	1	1	0.461
	gi|62701927	CBS domain, putative	3	2	2	1	1	0.534
	gi|50252988	unknown protein	4.3	4	4	1	1	0.549
	gi|125590644	hypothetical protein OsJ_15076	12	4	4	2	2	0.554
	gi|53749372	unknown protein	7.1	3	3	1	1	0.581
	gi|56784479	hypothetical protein	5.5	4	4	2	2	0.59
	gi|62701926	abscisic acid- and stress-induced protein	31.2	10	10	2	2	0.6
	gi|77553487	Nonspecific lipid-transfer protein 2 precursor, putative, expressed	27.4	10	10	2	2	1.506
	gi|19571117	OSJNBb0008G24.11	1.9	4	4	1	1	1.508
	gi|77548426	Nonspecific lipid-transfer protein precursor, putative, expressed	11	2	2	1	1	1.536
	gi|18461235	putative nuclear RNA binding protein A	7.1	7	7	3	3	1.761
	gi|70663913	OSJNBa0029H02.25	2.5	3	3	2	2	0.523
	gi|125602537	hypothetical protein OsJ_26407	20.9	2	2	1	1	1.58

^a^Cov indicates percentage of assigned peptides to the predicted protein.

^b^Spectrum indicates the number of assigned spectrum.

^c^Unique Spectrum indicates the number of assigned spectrum.

^d^Peptide indicates the number of assigned peptides.

^e^UniquePeptide indicates the number of assigned Unique peptides.

^f^The values were calculated as the ratio of salt stress treated protein content to the control.

### Classification of differentially expressed proteins

These differentially expressed proteins were enriched in terms for oxo-acid-lyase activity and tetrapyrrole binding based on GO molecular function enrichment analysis (S2 Table), and mainly in terms for nucleus, intracellular organelle part, chloroplast stroma, chromatin, photosystem I, and macromolecular complex based on GO cellular component enrichment analysis (S3 Table). GO biological process terms were enriched for cellular macromolecular complex subunit organization, nucleosome organization, cellular homeostasis, regulation of biological quality, chromatin organization, response to disaccharide stimulus, response to abiotic stimulus, multicellular organismal development, aromatic compound biosynthetic process, phenylpropanoid metabolic process, growth and post-embryonic development (S4 Table).

Using MapMan analysis, the major metabolic pathway was analyzed based on the differentially expressed proteins. The results indicated that nine of these proteins were engaged in the light reaction and mitochondrial electron transport (S1 Fig.). The regulation pathway was also analyzed by MapMan, and 11 differentially expressed proteins were involved in transcription, protein degradation, hormone metabolism, calcium signaling, G-protein signaling, and sugar and nutrient physiology signaling (Table 1, S2 Fig.). The U small nuclear ribonucleoprotein- (U-snRNP) and ribosome-related proteins were involved in RNA and protein synthesis (Table 1, S3 Fig.).

Furthermore, 16 notably differentially regulated proteins involved in the photosynthetic, antioxidant and oxidative phosphorylation pathways were further identified (Table 1).

#### Photosynthesis-related proteins

**Fig 3:**
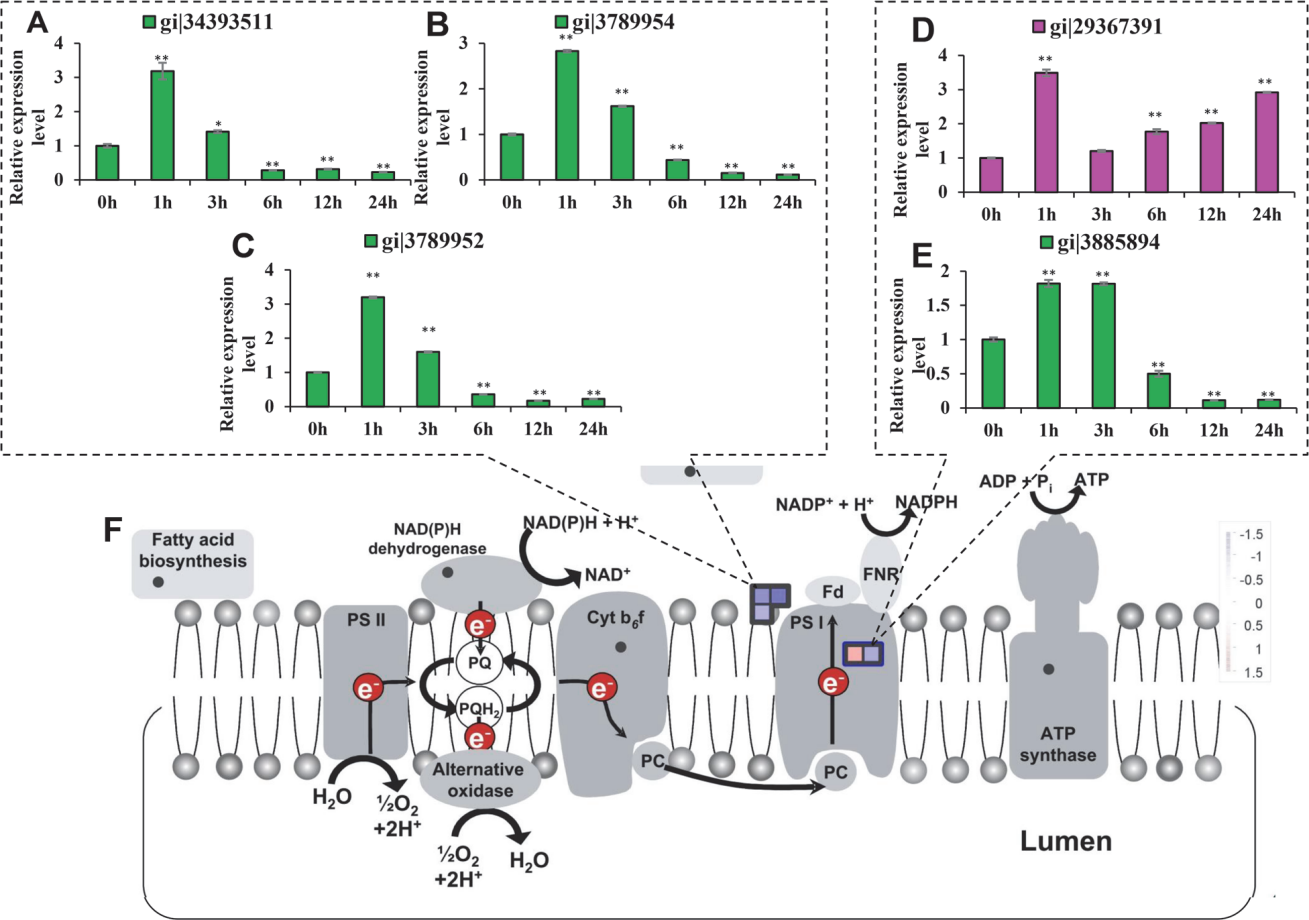
The differentially expressed proteins involved in photosynthesis. Transcript abundances of mRNAs encoding proteins involved in photosynthesis were analyzed at 0 h, 1 h, 3 h, 6 h, 12 h and 24 h following salt stress treatment. The mRNA levels at 12 h were compared with the iTRAQ data. Red indicates the proteins that were up-regulated while green indicates the proteins that were down-regulated. Significant differences were determined relative to each treatment using a student’s *t*-test [*P*-values <0.05 (*) and <0.01 (**)]. Bars: SD. The changes in transcript abundances at 12 h were compared with the iTRAQ data. *(A)* Putative PSI antenna protein (Lhca2, gi|34393511). *(B)* Chlorophyll a/b-binding protein precursor (Lhca1, gi|3789954). *(C)* Chlorophyll a/b-binding protein precursor (Lhca4, gi|3789952). *(D)* Chloroplast photosystem I reaction center subunit II precursor-like protein (PsaD, gi|29367391). *(E)* PSI H subunit GOS5 (PsaH, gi|3885894). *(F)* Overview of the differentially expressed proteins involved in photosynthesis.

Six differentially expressed proteins were involved in photosynthesis (Table 1, Fig. 3). Among three light-harvesting antenna complex I (LHCI) subunits, levels of the light-harvesting complex I subunit 1 (Lhca1) precursor, putative Lhca2 and Lhca4 precursors were reduced by 41.9%, 36.5% and 45.1% under salt stress treatment, respectively. The PSI subunit D (PsaD) precursor, chloroplast PSI reaction center subunit II precursor-like protein, was increased by 1.585-fold, while the levels of the PSI H subunit GOS5 reduced by 37.6%. The level of the putative chloroplast chaperonin was decreased by 34.9%. These proteins were only involved in PSI (S4 Fig.), and no protein organizing the PSII complex was found differently expressed in our data.

By qRT-PCR approach it was found that the expression patterns of the three Lhca mRNA transcripts were similar, which were increased at 1 h following salt stress treatment, then gradually decreased at 3 h and were significantly decreased after 6 h (Fig. 3A, 3B, 3C). The level of mRNA encoding PsaD was increased at 1 h following salt stress treatment, decreased at 3 h, and gradually increased from 6 h to 24 h (Fig. 3D). The level of the mRNA encoding PSI subunit H (PsaH) increased from 1 h to 3 h following salt stress treatment, and then gradually decreased from 6 h to 24 h (Fig. 3E). In comparison with the increased protein content of PsaD and decreased protein contents of PsaH and Lhcas after 24 h of salt stress treatment, the levels of the mRNAs encoding all of these proteins were in good agreement with the iTRAQ data from 6 h to 24 h.

**Fig 4:**
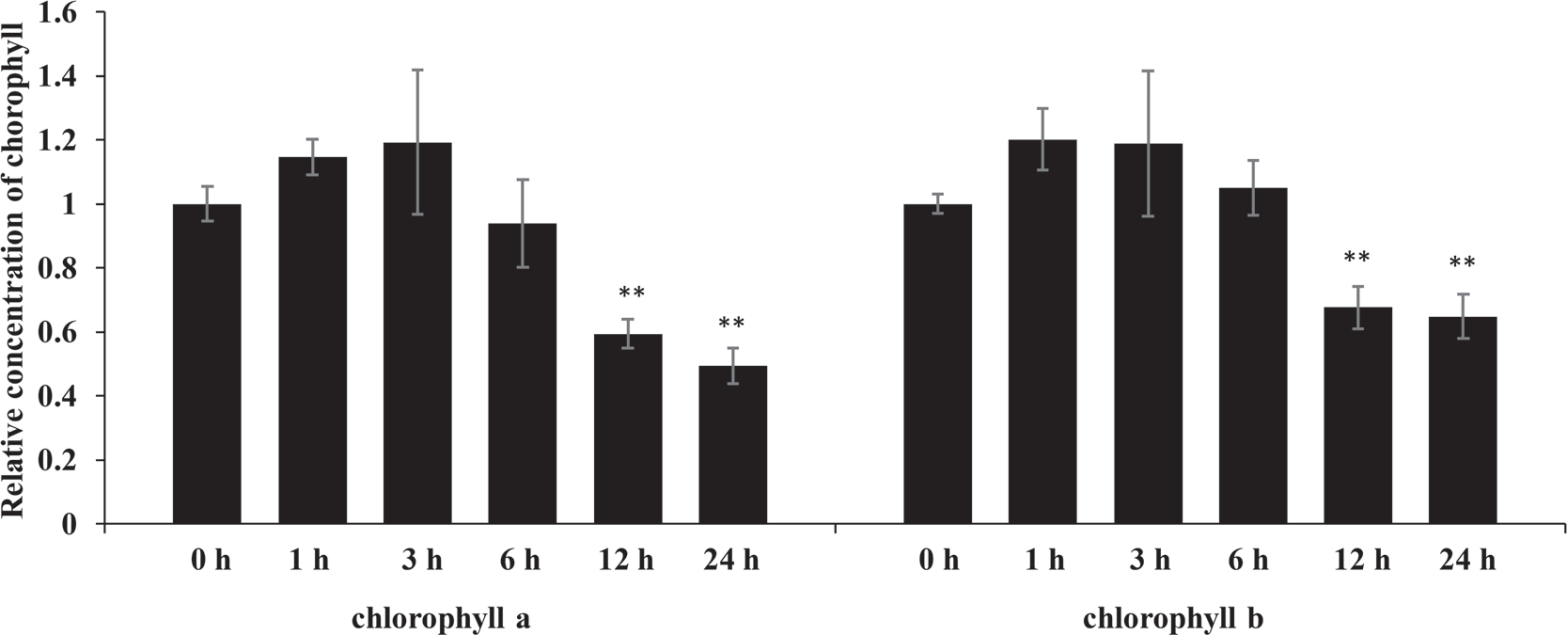
The chlorophyll a/b abundances in salt stress treated rice. The concentration of chlorophyll a/b in salt stress treated rice was analyzed at 0 h, 1 h, 3 h, 6 h, 12 h and 24 h following treatment. Significant differences were determined relative to each treatment using a student’s *t*-test [*P*-values <0.05 (*) and <0.01 (**)]. Bars: SD.

The physiological results showed that the chlorophyll a and chlorophyll b contents of rice shoots were significantly decreased after 12 h of salt stress treatment (Fig. 4). The significantly decreased levels of chlorophyll a and chlorophyll b were consistent with the changes in Lhca1, Lhca2 and Lhca4 levels in the iTRAQ data.

#### Antioxidant-related proteins

**Fig 5:**
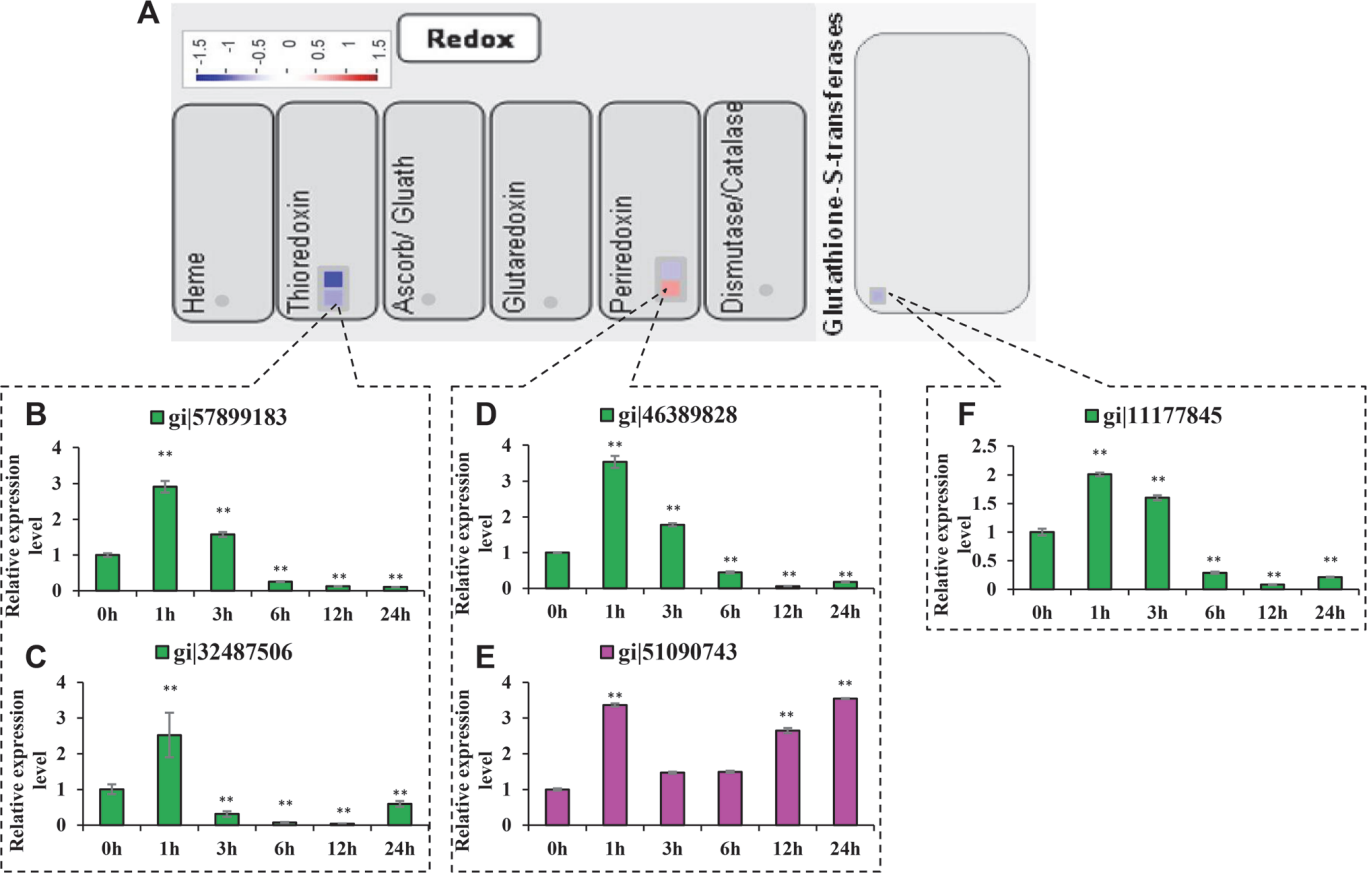
The differentially expressed proteins involved in the antioxidant pathway. Transcript abundances of mRNAs encoding antioxidant-related proteins were analyzed at 0 h, 1 h, 3 h, 6 h, 12 h and 24 h following salt stress treatment. The mRNA levels at 12 h were compared with the iTRAQ data. Red indicates the proteins that were up-regulated and green indicates the proteins that were down-regulated. Significant differences were determined relative to each treatment using a student’s *t*-test [*P*-values <0.05 (*) and <0.01 (**)]. Bars: SD. The changes in transcript abundances at 12 h were compared with the iTRAQ data. *(A)* Overview of differentially expressed proteins involved in antioxidant. *(B)* Thioredoxin M-like (gi|57899183). *(C)* TRX x (gi|32487506). *(D)* Putative thioredoxin peroxidase (gi|46389828). *(E)* Putative peroxiredoxin Q (gi|51090743). *(F)* Putative glutathione S-transferase OsGSTF3 (gi|11177845).

Five differentially expressed proteins were antioxidants (Table 1, Fig. 5). The levels of the thioredoxin M-like (TRX M-like), putative thioredoxin peroxidase (TPx), putative glutathione S-transferase F3 (GSTF3) and thioredoxin x (TRX x) proteins were reduced by 39.2%, 34%, 36.5% and 52.3% under salt stress treatment, respectively. The level of putative peroxiredoxin Q (PrxQ) protein was increased by 1.688-fold (Table 1, Fig. 5).

By qRT-PCR analysis it was found that the expression patterns of mRNAs encoding TRX M-like, TPx and GSTF3 proteins were similar, which were increased at 1 h of salt stress treatment, then gradually decreased at 3 h and significantly decreased after 6 h (Fig. 5B, 5D, 5F). The level of the mRNA encoding TRX x was increased 1 h after salt stress treatment, then significantly decreased after 3 h (Fig. 5C). The level of the mRNA encoding PrxQ was increased 1 h after salt stress treatment, and decreased from 3 h to 6 h, then again significantly increased from 12 h to 24 h (Fig. 5E). In comparison with the increased protein content of PrxQ and decreased protein contents of TRX M-like, TPx, GSTF3 and TRX x after 24 h of salt stress treatment, the levels of the mRNAs encoding these proteins were in good agreement with the iTRAQ data at 12 h and 24 h.

**Fig 6:**
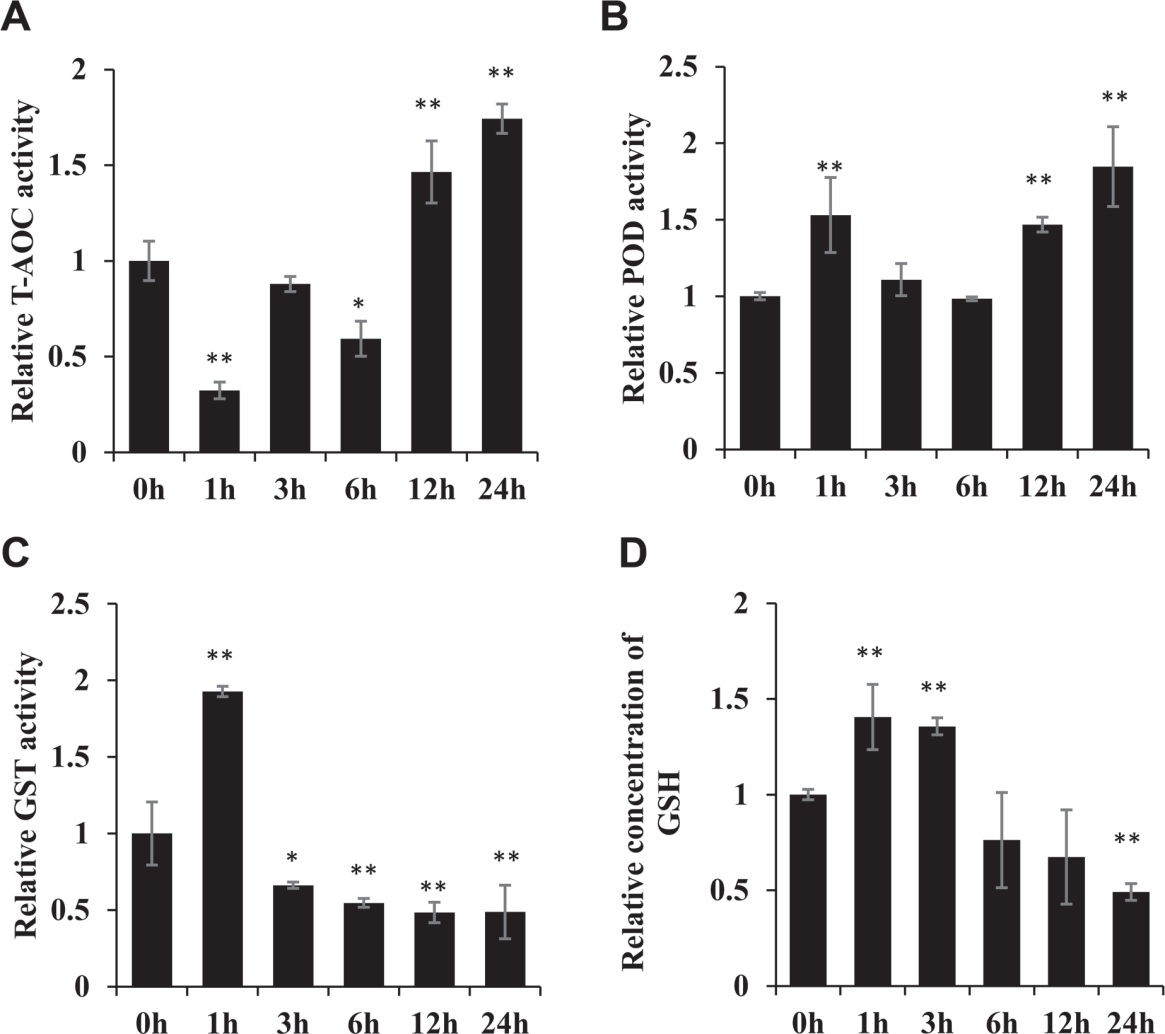
The antioxidant parameters in salt stress treated rice. Changes to rice antioxidant parameters were analyzed at 0 h, 1 h, 3 h, 6 h, 12 h and 24 h following salt stress treatment. Significant differences were determined relative to each treatment using a student’s *t*-test [*P*-values <0.05 (*) and <0.01 (**)]. Bars: SD. *(A)* The total antioxidant capacity (T-AOC) was analyzed in salt stress treated rice. *(B)* POD activity was analyzed in salt stress treated rice. *(C)* GST activity was analyzed in salt stress treated rice. *(D)* GSH concentrations were analyzed in salt stress treated rice.

The physiological data including T-AOC, POD activity, GST activity and GSH level were measured in salt stress treated rice shoots. It was found that the T-AOC and POD activity were both significantly increased at 12 h and 24 h following salt stress (Fig. 6A, 6B) which were consistent with the change in PrxQ level shown in the iTRAQ data. GST activity was increased 1 h after salt stress treatment and then significantly decreased (Fig. 6C). GSH content was increased in the first three hours and then significantly decreased at 24 h following salt stress treatment (Fig. 6D). Both the GST activity and the GSH content were significantly decreased 24 h following salt stress treatment, consistent with the change in GSTF3 level in the iTRAQ data.

#### Oxidative phosphorylation-related proteins

**Fig 7:**
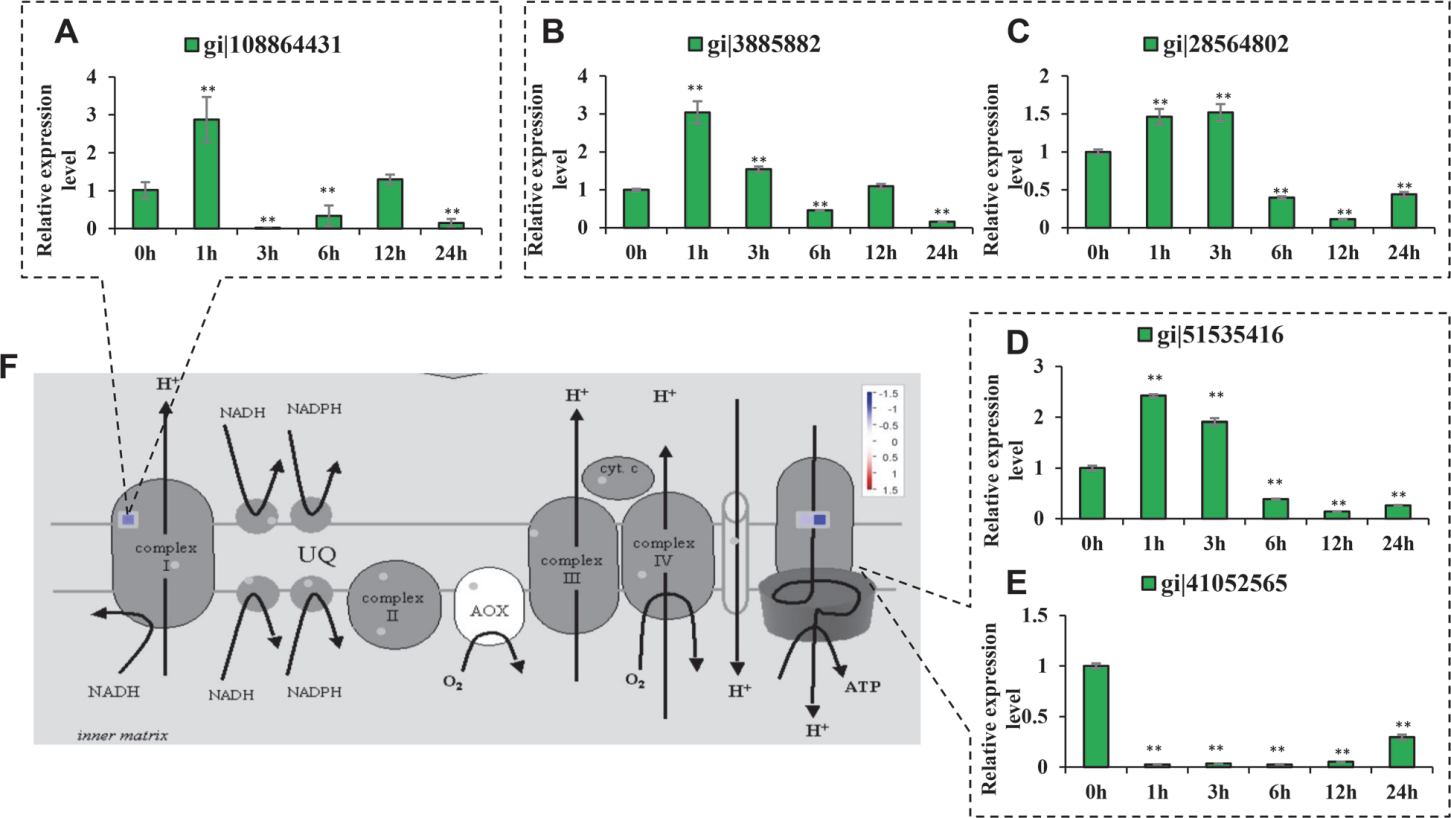
The differentially expressed proteins involved in oxidative phosphorylation. The transcript abundances of the mRNAs encoding proteins involved in oxidative phosphorylation were analyzed at 0 h, 1 h, 3 h, 6 h, 12 h and 24 h after salt stress treatment. The mRNA levels at 12 h were compared with the iTRAQ data. Red indicates the proteins that were up-regulated and green indicates the proteins that were down-regulated. Significant differences were determined relative to each treatment using a student’s *t*-test [*P*-values <0.05 (*) and <0.01 (**)]. Bars: SD. The changes in transcript abundances at 12 h were compared with the iTRAQ data. *(A)* Acyl carrier protein 2 (gi|108864431). *(B)* Inorganic pyrophosphatase (gi|3885882). *(C)* Putative vacuolar ATP synthase subunit H (gi|28564802). *(D)* Putative ATP synthase delta chain (gi|51535416). *(E)* Putative ATP synthase (gi|41052565). *(F)* Overview of the differentially expressed proteins involved in mitochondrial electron transport.

Five differentially expressed proteins were involved in oxidative phosphorylation in this report (Table 1, Fig. 7). The content of acyl carrier protein 2 was reduced by 68.1% under salt stress treatment, which is primarily involved in organizing the NADH dehydrogenase ND6/Ddufab1 subunits. The inorganic pyrophosphatase content was reduced by 37.7%. The levels of the putative vacuolar ATP synthase subunit H, ATP synthase delta chain and ATP synthase were reduced by 39.3%, 35.1% and 52%, respectively.

By qRT-PCR analysis it was found that the levels of the mRNAs encoding acyl carrier protein 2 and inorganic pyrophosphatase were decreased 6 h and 24 h after salt stress treatment, but not significantly different at 12 h (Fig. 7A, 7B). The levels of the mRNAs encoding vacuolar ATP synthase subunit H and ATP synthase delta chain were increased at 1 h and 3 h and then decreased from 6 h to 24 h (Fig. 7C, 7D). The level of the mRNA encoding ATP synthase was significantly down-regulated from 1 h to 24 h (Fig. 7E). In comparison with the decreased protein contents after 24 h of salt stress treatment, the levels of the mRNAs encoding vacuolar ATP synthase subunit H and ATP synthase delta chain were in good agreement with the iTRAQ data from 6 h to 24 h, and that encoding ATP synthase was in agreement from 1 h to 24 h. The levels of mRNAs encoding acyl carrier protein 2 and inorganic pyrophosphatase were only in agreement with iTRAQ data at 6 h and 24 h.

**Fig 8:**
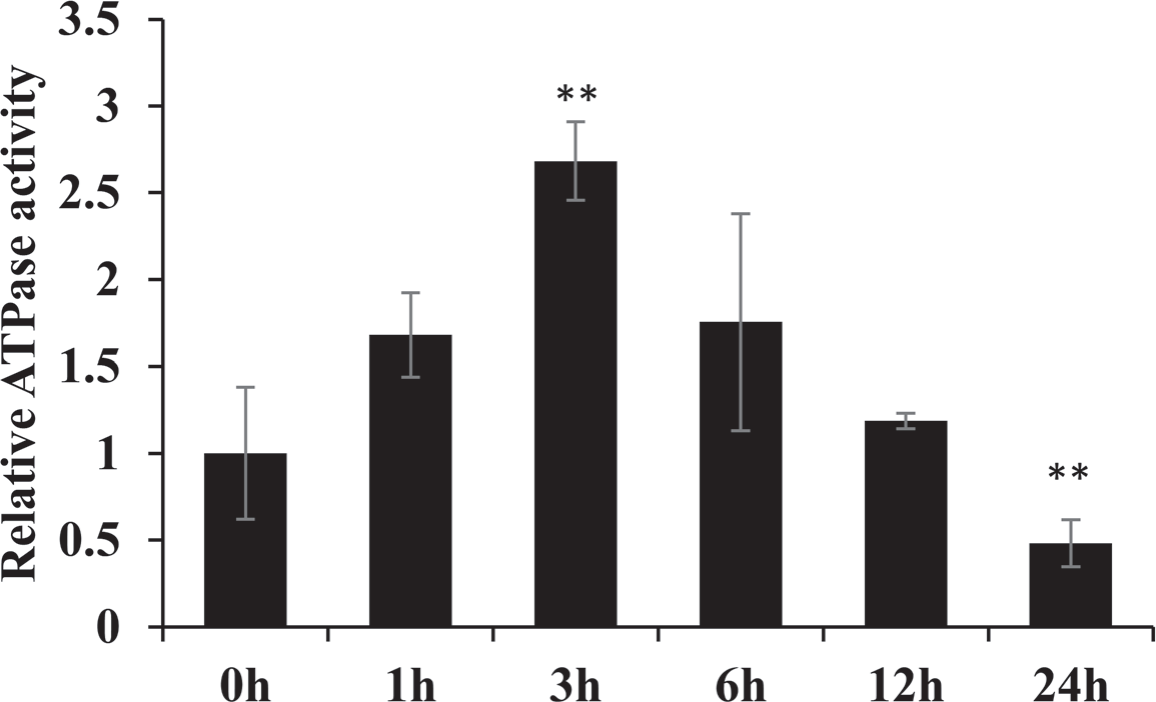
The ATPase activity assay. Changes in rice ATPase activity were assessed at 0 h, 1 h, 3 h, 6 h, 12 h and 24 h following salt stress treatment. Significant differences were determined relative to each treatment using a student’s *t*-test [*P*-values <0.05 (*) and <0.01 (**)]. Bars: SD.

To confirm the effects of salt stress on the energy supply for ionic regulation, the ATPase activity in salt stressed rice shoot was analyzed. It was found that the ATPase activity increased for the first three hours following salt stress treatment, then slightly decreased, and significantly decreased at 24 h following salt stress treatment (Fig. 8). This result was consistent with the alterations to proteins related to oxidative phosphorylation.

### Correlation of iTRAQ data with qRT-PCR

In addition to the mRNA levels of 15 differentially expressed proteins related to photosynthetic, antioxidant and oxidative phosphorylation pathways, 21 mRNAs encoding other proteins were randomly selected for analysis (S2, S3 and S5 Figs.). We compared the mRNA levels at 12 h with the iTRAQ data, and determined that approximately 86% of the qRT-PCR results were in good agreement with the iTRAQ data. The three exceptions were acyl carrier protein 2 (Fig. 7A), inorganic pyrophosphatase (Fig. 7B) and P-II nitrogen sensing protein (S2 Fig.).

**Fig 9:**
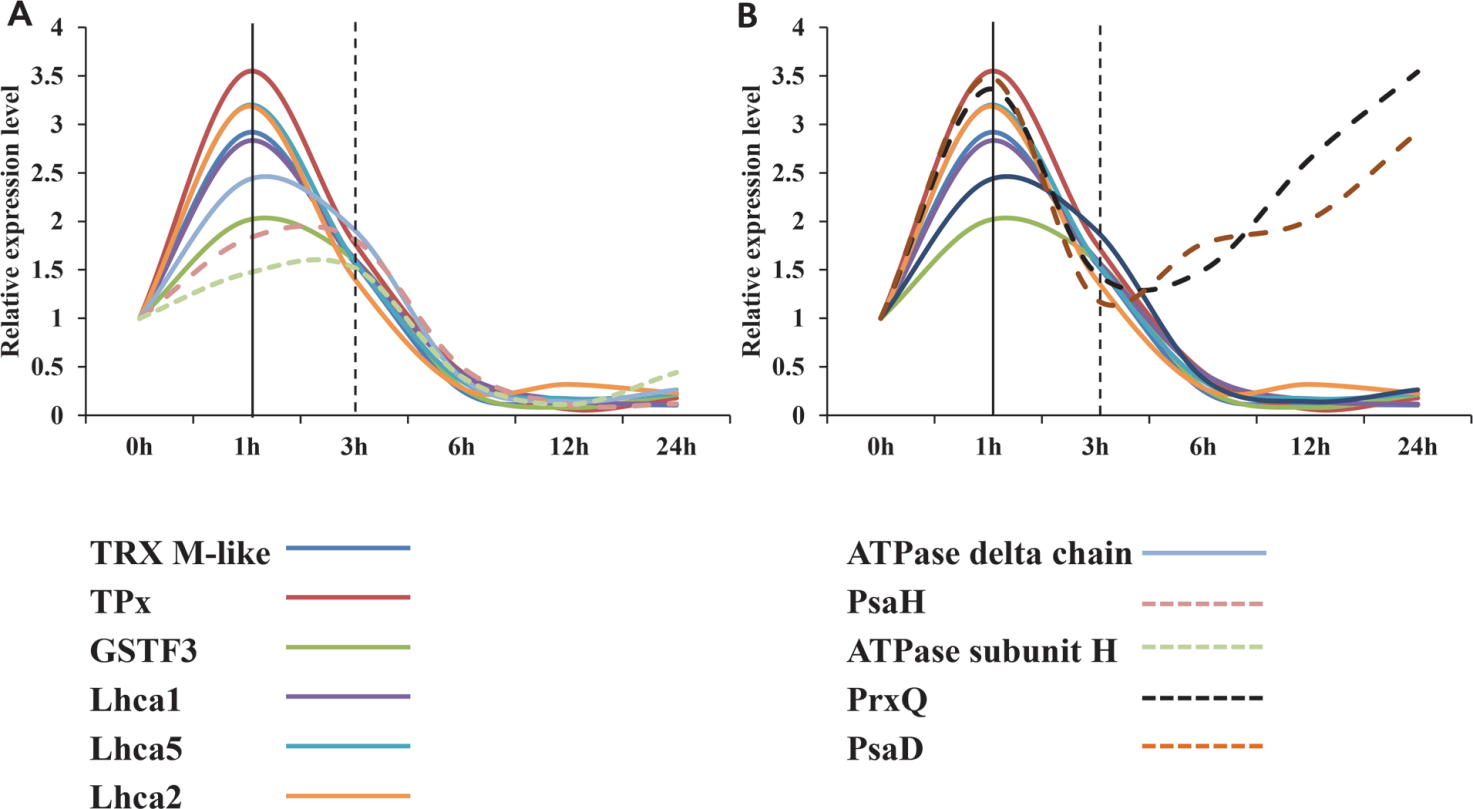
The expression patterns of genes encoding the differentially expressed proteins. The expression patterns of the genes encoding the differentially expressed proteins were compared. The flex points are indicated by the black lines. Different patterns are indicated by different types of lines. Different genes are indicated with different colors. *(A)* The similarly down-regulated patterns were compared. *(B)* The similar up-regulated patterns and down-regulated patterns were compared.

To verify the cooperation between the proteins, their expression patterns were compared (Fig. 9). As indicated by the qRT-PCR analysis, the expression patterns of mRNAs encoding TRX M-like, TPx, GSTF3, Lhca1, Lhca2, Lhca4 and ATPase delta chain were similar. Additionally, the expression patterns of mRNAs encoding PsaH and ATPase subunit H were similar to those patterns, with the exception of the delayed downregulation (Fig. 9A). We also showed that the expression patterns of mRNAs encoding PrxQ and PsaD contrasted with those of TRX M-like, TPx, GSTF3, Lhca1, Lhca2, Lhca4 and ATPase delta chain from 3 h to 24 h (Fig. 9B). These results indicate the potential for inter-related proteome reprogramming around PSI in rice shoots under salt stress.

## Discussion

Fifty six differentially expressed proteins were identified by quantitative proteomic analysis in this study. Two previous studies using 2-DE proteomic profiling of salt-stressed rice leaves identified 11 and 33 significantly regulated proteins respectively [18, 19]. Besides Ferritin and ATPase identified in all studies, many other salt-responsive proteins were newly identified in this research.

The previous proteomic studies also found that photosynthesis related proteins such as RuBisCO, chloroplast phosphoglycerate kinase and PSII oxygen evolving complex protein (OEP) were involved in the salt stress response[18, 19, 26]. Although RuBisCO subunits were not detected as being differentially expressed in our study, chloroplast chaperonin was found down-regulated by salt stress. Chloroplast chaperonin was reported to interact with RuBisCO [27] and it was demonstrated that the rice chloroplast chaperonin 60α subunit is required for the folding of the RuBisCO large subunit [28].

Under salt stress, photosynthetic ATP synthesis was reduced, inhibiting PSII [29]. It has been demonstrated that salt stress itself has no direct effect on PSII activity but blocks electron transport between QA and QB [13, 14], potentially explaining why differentially expressed PSII-related proteins were not detected in our study. Antioxidant related proteins were also detected in previous proteomic studies. In suspension-cultured rice cells under salt stress, GST and glutaredoxin (GRX) contents were down-regulated; however, no change in TRX levels was observed [22]. Similarly, putative TPx, TRX M-like, putative GSTF3 and TRX x proteins were found down-regulated in our study. We determined that salt stress affected several pathways, including the antioxidant, photosynthesis and oxidative phosphorylation. Interestingly, these pathways are inter-connected and PSI plays an important role in the salt stress response.

### The different response of PSI subunits to salt stress

It was previously reported the gradually decreasing activities of PSI and PSII with increasing salt concentrations [12], but it has not been documented the mechanism of PSI salt-response in plants and also not clear for the responses of PS I reaction center proteins to salt stress or oxidative stress. This study was helpful to get insight into the mechanism of photosynthesis salt-response.

PsaD level was increased under salt stress while the levels of PsaH, Lhca1, Lhca2 and Lhca4 were decreased (Fig. 3, Table 1), which seemed controversy; however, the opposite alteration of PsaD was reasonable based on the previous studies. As it was reported, PSI activity is increased in *Cyanobacteria* under salt stress [15], while in rice, it gradually decreases with increasing salt concentration [12]. PsaH is only found in plants and green algae, and can bind all five Lhca proteins [30]. Furthermore, LHCI is absent in *Cyanobacteria* [16]; therefore, the conflict may arise from the different salt stress response mechanisms that were developed during evolution. In addition, PSI devoid of PsaH has only 61% of the wild type NADP^+^ photoreduction activity in *Arabidopsis* [31], and downregulation of PsaH in rice shoots would thus inhibit NADPH generation and decrease PSI activity. Several lines of evidence have implicated the Fd-docking function of PsaD but have not demonstrated a functional requirement of PsaD in NADP^+^ photoreduction [16]. With regards to Fd’s role in electron flow [17], PsaD upregulation might contribute to the enhanced cyclic electron flow around PSI, which would improve ATP production but not affect the PSI activity. Therefore, the controversy was interesting, and it gave some clue on the role of PsaD in cyclic electron flow and the reason for the opposite responses of PS I in *Cyanobacteria* and plant.

**Fig 10:**
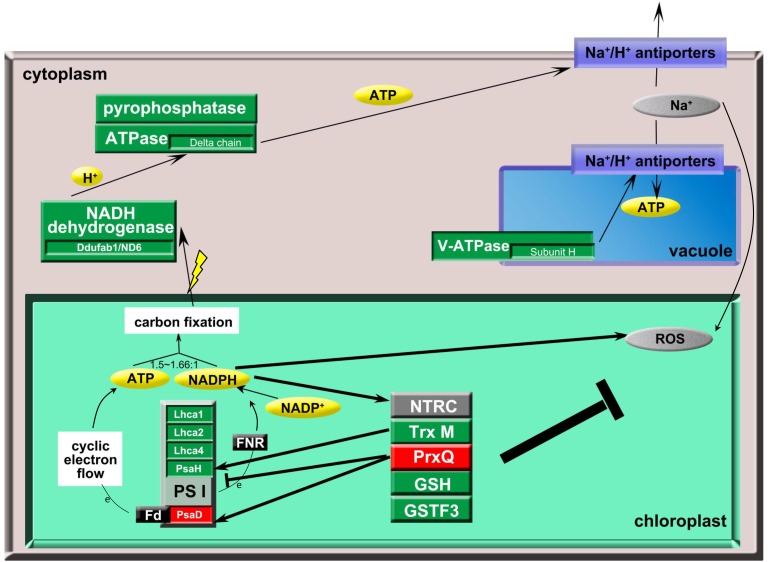
The coordinated reprogramming of the rice proteome under salt stress. Under salt stress, the responses of the proteins involved in antioxidant, photosynthetic and oxidative phosphorylation pathways are inter-connected and PSI plays an important role in the salt stress response. The response of antioxidant-related proteins regulates the photosystem, and PSI plays an important role in maintaining the energy supply for ionic regulation. To maintain the ATP/NADPH ratio for carbon fixation, PsaH is down-regulated to reduce NADPH production, and Fd-docking functional PsaD is up-regulated to increase ATP production by enhancing the cyclic electron flow. In the diagram, red indicates the proteins that were up-regulated, green indicates the proteins that were down-regulated, and black lines indicate the relationship between the proteins.

During carbon fixation, the required ATP/NADPH ratio varies from 1.5 to 1.66 [32], and the reduced NADPH inhibits NADPH oxidase function and reduce ROS formation. To maintain the ATP/NADPH ratio for carbon fixation and prevent the production of extra NADPH for ROS generation, the plant-specific PsaH was down-regulated to reduce NADPH production, and Fd-docking functional PsaD was up-regulated to increase ATP production by enhancing the cyclic electron flow (Fig. 10).

### The response of antioxidant-related proteins to salt stress

PrxQ, TRX M-like, TRX x, TPx, and GSTF3 may play important role as antioxidants in plant responses to salt stress. Although TPx, TRX M-like, TRX x and GSTF3 were reduced by salt treatment, the T-AOC was increased. On the one hand, TRX, glutathione and ascorbic acid (AsA) play a part in redox homeostasis while POD and CAT participate in the scavenging of ROS [33]. The increased T-AOC might be mainly determined by the response of PrxQ. On the other hand, besides those antioxidant-related proteins, malondialdehyde (MDA), reduced state of AsA and other members of TRX family also determine the redox potential [34]. As reported previously, PrxQ functions as a monomeric protein and represents about 0.3% of chloroplast proteins. PrxQ is reductively regenerated by various TRX proteins and the reduction efficiency of TRX was established as AtTrx y1 > AtTrx y2 > AtTrx x > AtTrx m4 [35]. Therefore, PrxQ was directly related to ROS homeostasis generated in chloroplast.

PrxQ also plays a role in regulating other antioxidant-related proteins. As reported previously, *PrxQ* deficiency can up-regulate the levels of mRNAs encoding glutathione reductase and glutathione synthetase [35], thus decreasing GSTF3 and GSH contents, and the GST activities observed in this research might be regulated by PrxQ. Furthermore, alterations to GST and GSH levels might affect PSI via their functions in hydrogen peroxide degradation and photosystem protection [6, 36].

Besides PrxQ, TPx also can eliminate excessively accumulated hydrogen peroxide in chloroplasts, and the reduction primarily relies on NADPH-TRX reductase C (NTRC) and TRXs [37]. So the downregulation of PsaH would inhibit the function of NTRC for the role of PsaH in NADPH generation. Accompanying the decreased TRX contents, the alteration in PsaH contents would ultimately inhibit TPx function.

### The relationship between antioxidants and photosystem under salt stress

ROS and antioxidants including PrxQ, TRX M-like, TRX x, TPx, and GSTF3 are differentially affected by salt stress and act as signaling factors regulating the response of PSI. First of all, PSII is protected by PrxQ, and deficiency of *PrxQ* down-regulates the expression of *PsbB* and *PsbH* but up-regulates the expression of *PsaA* and *PsaJ* in *Arabidopsis* [35]. It was likely that the additional PrxQ could also regulate PSI subunits including PsaD and PsaH as our data showed. In addition, TRXs can regulate photosynthesis and chloroplast gene expression [37–39]. Consequently, the decreased TPx, TRX M-like and TRX x expression might also modulate PsaD, PsaH and Lhca function.

Together with the increased PrxQ under salt stress, the decreased TRX M-like, TRX x, TPx and GSTF3 might down-regulate PsaH and up-regulate PsaD to balance carbon fixation and ROS generation. Alternatively, changes in NADPH production in PSI could affect NTRC function in hydrogen peroxide reduction (Fig. 10).

### The role of PSI in maintaining the energy supply for ionic regulation

In plants, ATPase, pyrophosphatase and vacuolar ATPase supply energy for Na^+^ transport into the vacuole and other tissues through Na^+^/H^+^ anti-porters [40, 41]. Consequently, changes in the levels of these proteins would lead to the excessive cellular accumulation of Na^+^. Additionally, the effects of salt stress on PSII would reduce carbon fixation and ultimately limit the energy for ionic regulation; however, alterations to PSI could balance the ATP/NADPH for carbon fixation and protect PSII from excessive excitation energy under salt stress [16]. As a result, the changes in PsaD and PsaH levels play an important role in maintaining the energy supply for ionic regulation (Fig. 10).

## Conclusions

By quantitative proteomic analysis of the rice salt response, 56 differentially expressed proteins in shoot were identified in this study. The physiological and qRT-PCR results were consistent with the proteomic data. Among these proteins, PrxQ and PsaD were up-regulated, while TRX M-like, TRX x, TPx, GSTF3, Lhcas, PsaH, chloroplast chaperonin, vacuolar ATP synthase subunit H, ATP synthase, and ATP synthase delta chain were down-regulated upon salt stress. These proteins were involved in antioxidant, photosynthetic and oxidative phosphorylation pathways. Importantly, our study suggests the inter-connection of these pathways and important role of PSI and PrxQ in the salt responses. Quantitative proteomic analysis is useful in revealing salt stress response mechanism and critical proteins although the precise functions of those proteins remain to be further elucidated.

## Supporting Information

S1 Table The primers for qRT-PCR. (DOC)

S2 Table GO molecular function enrichment analysis of the differentially expressed proteins. (DOC)

S3 Table GO cellular component enrichment analysis of the differentially expressed proteins. (DOC)

S4 Table GO biological process enrichment analysis of the differentially expressed proteins. (DOC)

S5 Table The information of spectrums for each differentially expressed proteins. (DOC)

S6 Table The homologue of the differentially expressed proteins. (DOC)

S1 Fig Metabolic pathway overview. (TIF)

S2 Fig The differentially expressed proteins involved in regulation. The transcript abundances of mRNAs encoding proteins involved in regulation were analyzed at 0 h, 1 h, 3 h, 6 h, 12 h and 24 h following salt stress treatment. The mRNA levels at 12 h were compared with the iTRAQ data. Red indicates the proteins that were up-regulated and green indicates the proteins that were down-regulated. Significant differences were determined relative to each treatment using a student’s *t*-test [*P*-values <0.05 (*) and <0.01 (**)]. Bars: SD. The changes in transcript abundances at 12 h were compared with the iTRAQ data. *(A)* Overview of differentially expressed proteins involved in regulation. *(B)* Putative MAR binding filament-like protein 1 (gi|55296302). *(C)* Putative transcription factor (gi|24431603). *(D)* Carboxyvinyl-carboxyphosphonate phosphorylmutase (gi|77553225). *(E)* 26S proteasome regulatory particle triple-A ATPase subunit 4 (gi|11094192). *(F)* Auxin-repressed protein-like protein ARP1 (gi|77552436). *(G)* Putative CBS domain containing protein (gi|34015153). *(H)* Hydroperoxide lyase (gi|33358444). *(I)* Putative P-II nitrogen sensing protein (gi|50878396).
(TIF)

S3 Fig The differentially expressed proteins involved in RNA and protein synthesis. The transcript abundances of mRNAs encoding proteins involved in RNA and protein synthesis were analyzed at 0 h, 1 h, 3 h, 6 h, 12 h and 24 h following salt stress treatment. The mRNA levels at 12 h were compared with the iTRAQ data. Red indicates the proteins that were up-regulated and green indicates the proteins that were down-regulated. Significant differences were determined relative to each treatment using a student’s *t*-test [*P*-values <0.05 (*) and <0.01 (**)]. Bars: SD. The changes in transcript abundances at 12 h were compared with the iTRAQ data. *(A*) Overview of differentially expressed proteins involved in RNA and protein synthesis. *(B)* Putative ribosomal protein L34 (gi|37805854). *(C)* Putative acidic ribosomal protein P1a (gi|50725625). *(D)* 26S proteasome regulatory particle triple-A ATPase subunit 4 (gi|11094192).
(TIF)

S4 Fig Overview of photosynthesis. (TIF)

S5 Fig The qRT-PCR analysis of the mRNAs encoding the differentially expressed proteins. The transcript abundances of mRNAs encoding the differentially expressed proteins were analyzed at 0 h, 1 h, 3 h, 6 h, 12 h and 24 h following salt stress treatment. The mRNA levels at 12 h were compared with the iTRAQ data. Red indicates the proteins that were up-regulated and green indicates the proteins that were down-regulated. Significant differences were determined relative to each treatment using a student’s *t*-test [*P*-values <0.05 (*) and <0.01 (**)]. Bars: SD. The changes in transcript abundances at 12 h were compared with the iTRAQ data. *(A)* qRT-PCR analysis of mRNA encoding H2A protein (gi|6319146). *(B)* qRT-PCR analysis of mRNAs encoding up-regulated proteins OSJNBb0008G24.11 (gi|19571117), putative nuclear RNA binding protein A (gi|18461235), Actin (gi|34851127), putative Isocitrate lyase (gi|34393921) and nonspecific lipid-transfer protein 2 precursor (gi|77553487). *(C)* qRT-PCR analysis of mRNAs encoding down-regulated proteins CBS domain (gi|62701927), OSJNBa0029H02.25 (gi|70663913), putative isopentenyl pyrophosphate: dimethyllallyl pyrophosphate isomerase (gi|27260946) and ferritin (gi|21686526).
(TIF)
