# Supplementary material for: Quantitative Proteomic Analysis of the Rice (Oryza sativa L.) Salt Response
Source: PLoS One. 2015 Mar 20;10(3):e0120978. doi: 10.1371/journal.pone.0120978 (PMC4368772; doi:10.1371/journal.pone.0120978)
Supplement: S1 Table — (DOC) [file pone.0120978.s001.doc]

# **S1 Table. The primers for qRT-PCR.**

| genes | primers | sequence |
| --- | --- | --- |
| *18S rRNA* | Forward primer(5'-3') | ATGGTGGTGACGGGTGAC |
|  | Reverse primer(5'-3') | CAGACACTAAAGCGCCCGGTA |
| gi|77552436 | Forward primer(5'-3') | TGTTGAGTGCGTGGAAGCAGAG |
|  | Reverse primer(5'-3') | GCCGTCGCCATCTTTGTTGATG |
| gi|18461235 | Forward primer(5'-3') | TGAGATGAAGCGTGATGGTGCAG |
|  | Reverse primer(5'-3') | TCAAGGCTTCCTCAGTCTCCTG |
| gi|34851127 | Forward primer(5'-3') | AAGGCTAACAGGGAGAAGATGACC |
|  | Reverse primer(5'-3') | CACCAGAGTCCAACACGATACCAG |
| gi|51090743 | Forward primer(5'-3') | AAGAACGGCGTCGTCCAGTACATC |
|  | Reverse primer(5'-3') | GATCTTGAGGGTCTCGCCAATGTG |
| gi|55296302 | Forward primer(5'-3') | GATCTCGCAACGGAGACAAAGG |
|  | Reverse primer(5'-3') | TGGTTGCAAAGCTTCAGGTAGAGG |
| gi|34393921 | Forward primer(5'-3') | AAGGAGTTACTGAAGAGC |
|  | Reverse primer(5'-3') | TGGATTTGGCAAGAACAT |
| gi|24431603 | Forward primer(5'-3') | TGGAACACCACAGACGAAGAAGC |
|  | Reverse primer(5'-3') | TTCTGGAACTGCTCAGCAAGCC |
| gi|29367391 | Forward primer(5'-3') | GTCAAGTTCACCGGCAAGAACGTC |
|  | Reverse primer(5'-3') | AACTGAAGAGCAGCCTGACAGATG |
| gi|37805854 | Forward primer(5'-3') | AATTCCACACCTGAGACCTGCTG |
|  | Reverse primer(5'-3') | TTCACGGTCCTGCGATTCCTTG |
| gi|19571117 | Forward primer(5'-3') | ATCCAAAGACGACGCTCTTGCC |
|  | Reverse primer(5'-3') | AGATCACCGCGTAGATGATGCAC |
| gi|77553487 | Forward primer(5'-3') | TGCTCCAGCGTGAACTAATCCG |
|  | Reverse primer(5'-3') | ATACAAAGCGTGAGACGACAGC |
| gi|34015153 | Forward primer(5'-3') | TGACAGTTCAGGCAAATTGGTTGG |
|  | Reverse primer(5'-3') | TGTCTCCAAAGGGTCAATCCACAG |
| gi|46389828 | Forward primer(5'-3') | AGCTCTCGTAGGTGGCAACAATG |
|  | Reverse primer(5'-3') | AACGACGACGCTACTCACCAAC |
| gi|41052905 | Forward primer(5'-3') | GGAAACCTTGTCATCGCTTTGAGG |
|  | Reverse primer(5'-3') | TCCAAAGCACTCTCAATCTGTTGG |
| gi|41052565 | Forward primer(5'-3') | TCGACACCTTTACAAAGGGCATCC |
|  | Reverse primer(5'-3') | AGGCCACTCTTTATCCTGATCTCC |
| gi|32487506 | Forward primer(5'-3') | AGGTACCAGGGAGCAGAAGAGAAG |
|  | Reverse primer(5'-3') | CTCGAACCTCTCTTGTCAACCTCA |
| gi|27260946 | Forward primer(5'-3') | TGCTGCTCAGAGGAAGCTCTTG |
|  | Reverse primer(5'-3') | ACGATGAACAGCAGGTAGTCAAGC |
| gi|3789952 | Forward primer(5'-3') | ATTTCATCAGGGCCAGGGCATC |
|  | Reverse primer(5'-3') | TCGCCCAATCATCGGTTTCACC |
| gi|11177845 | Forward primer(5'-3') | TCTTCGAGTCGAGGGCTATTTGC |
|  | Reverse primer(5'-3') | TGCCCATGAGGGTCTTGTTACC |
| gi|33358444 | Forward primer(5'-3') | AGTACGTGTACTGGTCCAACGG |
|  | Reverse primer(5'-3') | GAACAAACAGTGTGCCGCCAAG |
| gi|77553225 | Forward primer(5'-3') | GCATGGCCAAAGAAGTGTGGAC |
|  | Reverse primer(5'-3') | AGTCCGGGCCACGATAAAGAAG |
| gi|21686526 | Forward primer(5'-3') | GTGATACCAGCAGAGGAG |
|  | Reverse primer(5'-3') | ACGATAAAGAAGTCCCGAT |
| gi|70663913 | Forward primer(5'-3') | CTGACGCATCGTCTGATATGCC |
|  | Reverse primer(5'-3') | AGTGCCACGATGTTTCCCACTG |
| gi|28564802 | Forward primer(5'-3') | AGCCAACGAGGTCCTTTGCTTG |
|  | Reverse primer(5'-3') | TGCTGCTCGTTTAGGATTTACTGC |
| gi|3885882 | Forward primer(5'-3') | TCATCCATGGCACGATCTGGAG |
|  | Reverse primer(5'-3') | TGCCTCTAGGAATTTCAACCACAC |
| gi|57899183 | Forward primer(5'-3') | AACATCACCTTCACCGCCAAGGAC |
|  | Reverse primer(5'-3') | TCATGAACCTGACCCTGCTTCCTC |
| gi|6319146 | Forward primer(5'-3') | TTCCCTGTTGGCCGTATCCATC |
| /Os03g0743400 | Reverse primer(5'-3') | CTCAGGCAAATGGACGTGTTGGT |
| gi|6319146 | Forward primer(5'-3') | ACAAGACCTCCAAAGAGTGATGCG |
| /Os03g0162200 | Reverse primer(5'-3') | CCTCATGTGCTTTGCTTGACTCGA |
| gi|6319146 | Forward primer(5'-3') | ACCCTCATCAAGGGCACCATAG |
| /Os10g0418000 | Reverse primer(5'-3') | GCACATCCACAAGTCCCTCATC |
| gi|50878396 | Forward primer(5'-3') | CAACTGAGAGGCATGAAGG |
|  | Reverse primer(5'-3') | GACACCACTATTTCCATCTTAACT |
| gi|11094192 | Forward primer(5'-3') | TCGGGAACTCGTGTTGTTCTTGAC |
|  | Reverse primer(5'-3') | TAAACCACAGGGTCCACCTCAC |
| gi|108864431 | Forward primer(5'-3') | TCTTCTTGCTCTGAAACCATCGC |
|  | Reverse primer(5'-3') | GAAGAGACGTCAGTCTAGCTAGCT |
| gi|3885894 | Forward primer(5'-3') | GTCTACTTCGACCTCGAGGACATC |
|  | Reverse primer(5'-3') | ACGTCTCGAAGAACTTGCTCTGG |
| gi|62701927 | Forward primer(5'-3') | CGGCATCGTCACCGATAAAGAC |
|  | Reverse primer(5'-3') | ATTGTTTGCTCAACCCGCAACC |
| gi|51535416 | Forward primer(5'-3') | CGACCGGGAAGGATATCAAGGTTC |
|  | Reverse primer(5'-3') | TTGGCCGCTGTAAGGAACAGAG |
| gi|3789954 | Forward primer(5'-3') | AGGAGATCAAGAACGGGCGATTG |
|  | Reverse primer(5'-3') | GATGTCGCCGATGTTGTTGTGC |
| gi|50725625 | Forward primer(5'-3') | AGCGACGATGACATGGGTTTCAG |
|  | Reverse primer(5'-3') | CCAGTCCGCCTATGCTTTGCAA |
| gi|34393511 | Forward primer(5'-3') | TCAGTGTTCAGAGGCAGTTTGCAG |
|  | Reverse primer(5'-3') | AAGCTGCATTCGCTGGTCTCAC |
