# Supplementary material for: Quantitative Proteomic Analysis of the Rice (Oryza sativa L.) Salt Response
Source: PLoS One. 2015 Mar 20;10(3):e0120978. doi: 10.1371/journal.pone.0120978 (PMC4368772; doi:10.1371/journal.pone.0120978)
Supplement: S2 Table — (DOC) [file pone.0120978.s002.doc]

# **S2 Table. GO molecular function enrichment analysis of the differentially expressed proteins.**

| GO term | Proteins | P-value |
| --- | --- | --- |
| oxo-acid-lyase activity | gi|77553225, gi|34393921 | 0.005281017 |
| tetrapyrrole binding | gi|34393511, gi|3789954, gi|3789952 | 0.01131486 |
