# Supplementary material for: Quantitative Proteomic Analysis of the Rice (Oryza sativa L.) Salt Response
Source: PLoS One. 2015 Mar 20;10(3):e0120978. doi: 10.1371/journal.pone.0120978 (PMC4368772; doi:10.1371/journal.pone.0120978)
Supplement: S3 Table — (DOC) [file pone.0120978.s003.doc]

# **S3 Table. GO cellular component enrichment analysis of the differentially expressed proteins.**

| GO term | Proteins | P-value |
| --- | --- | --- |
| nucleus | gi|50252685, gi|41052905, gi|41052565, gi|11094192, gi|37805854, gi|125590644, gi|113578236, gi|25553579, gi|34851127, gi|6319146 | 0.000303 |
| intracellular organelle part | gi|34393511, gi|41052905, gi|3789954, gi|51090743, gi|3885882, gi|51535416, gi|28564802, gi|125590644, gi|77553225, gi|3885890, gi|56784479, gi|12039318, gi|11974, gi|3789952, gi|29367391, gi|55296302, gi|50252685, gi|3885894, gi|41052565, gi|32487506, gi|11094192, gi|18855008, gi|37805854, gi|113578236, gi|57899183, gi|46389828, gi|25553579, gi|34015153, gi|6319146, gi|34851127 | 0.001116 |
| chloroplast stroma | gi|29367391, gi|3885894, gi|3789954, gi|51090743, gi|3789952 | 0.001473 |
| chromatin | gi|3885890, gi|12039318, gi|6319146 | 0.001502 |
| photosystem I | gi|29367391, gi|3885894, gi|34393511 | 0.002202 |
| chromosomal part | gi|3885890, gi|12039318, gi|6319146 | 0.003076 |
| photosystem | gi|29367391, gi|3885894, gi|34393511, gi|3789954, gi|3789952 | 0.006638 |
| organelle part | gi|34393511, gi|41052905, gi|3789954, gi|51090743, gi|3885882, gi|51535416, gi|28564802, gi|125590644, gi|77553225, gi|3885890, gi|56784479, gi|12039318, gi|11974, gi|3789952, gi|29367391, gi|55296302, gi|50252685, gi|3885894, gi|41052565, gi|32487506, gi|11094192, gi|18855008, gi|37805854, gi|113578236, gi|57899183, gi|46389828, gi|25553579, gi|34015153, gi|6319146, gi|34851127 | 0.008763 |
| macromolecular complex | gi|34393511, gi|41052905, gi|50725625, gi|3789954, gi|51535416, gi|28564802, gi|125590644, gi|56784479, gi|11974, gi|3789952, gi|29367391, gi|50252685, gi|3885894, gi|11094192, gi|37805854, gi|113578236, gi|14495192, gi|25553579 | 0.009445 |
| chromosome | gi|3885890, gi|12039318, gi|6319146 | 0.014908 |
| photosynthetic membrane | gi|29367391, gi|3885894, gi|34393511, gi|3789954, gi|3789952 | 0.015935 |
| protein complex | gi|29367391, gi|3885894, gi|34393511, gi|3789954, gi|11094192, gi|51535416, gi|28564802, gi|125590644, gi|14495192, gi|56784479, gi|3789952 | 0.018818 |
| nucleoplasm | gi|113578236, gi|41052905 | 0.024439 |
| intracellular membrane-bounded organelle | gi|53749372, gi|34393511, gi|50725625, gi|3789954, gi|51535416, gi|50252685, gi|62701927, gi|3885894, gi|34393921, gi|108864431, gi|11094192, gi|18855008, gi|50878396, gi|125600465, gi|34851127, gi|6319146, gi|41052905, gi|51090743, gi|3885882, gi|28564802, gi|21686526, gi|77553225, gi|125590644, gi|56784479, gi|3885890, gi|12039318, gi|3789952, gi|11974, gi|29367391, gi|55296302, gi|50252988, gi|41052565, gi|32487506, gi|27260946, gi|5922611, gi|37805854, gi|113578236, gi|57899183, gi|46389828, gi|14495192, gi|25553579, gi|34015153 | 0.030583 |
| plastid thylakoid | gi|29367391, gi|55296302, gi|3885894, gi|34393511, gi|3789954, gi|32487506, gi|51090743, gi|57899183, gi|56784479, gi|3789952 | 0.035712 |
| organelle subcompartment | gi|29367391, gi|55296302, gi|3885894, gi|34393511, gi|3789954, gi|32487506, gi|51090743, gi|57899183, gi|56784479, gi|3789952 | 0.037015 |
| membrane-bounded organelle | gi|53749372, gi|34393511, gi|50725625, gi|3789954, gi|51535416, gi|50252685, gi|62701927, gi|3885894, gi|34393921, gi|108864431, gi|11094192, gi|18855008, gi|50878396, gi|125600465, gi|34851127, gi|6319146, gi|41052905, gi|51090743, gi|3885882, gi|28564802, gi|21686526, gi|77553225, gi|125590644, gi|56784479, gi|3885890, gi|12039318, gi|3789952, gi|11974, gi|29367391, gi|55296302, gi|50252988, gi|41052565, gi|32487506, gi|27260946, gi|5922611, gi|37805854, gi|113578236, gi|57899183, gi|46389828, gi|14495192, gi|25553579, gi|34015153 | 0.03781 |
| thylakoid | gi|29367391, gi|55296302, gi|3885894, gi|34393511, gi|3789954, gi|32487506, gi|51090743, gi|57899183, gi|56784479, gi|3789952 | 0.038352 |
