# Supplementary material for: Quantitative Proteomic Analysis of the Rice (Oryza sativa L.) Salt Response
Source: PLoS One. 2015 Mar 20;10(3):e0120978. doi: 10.1371/journal.pone.0120978 (PMC4368772; doi:10.1371/journal.pone.0120978)
Supplement: S4 Table — (DOC) [file pone.0120978.s004.doc]

# **S4 Table. GO biological process enrichment analysis of the differentially expressed proteins.**

| GO term | Proteins | P-value |
| --- | --- | --- |
| cellular macromolecular complex subunit organization | gi|113578236, gi|3885890, gi|56784479, gi|12039318, gi|6319146 | 0.002203095 |
| nucleosome organization | gi|3885890, gi|12039318, gi|6319146 | 0.003972202 |
| macromolecular complex subunit organization | gi|113578236, gi|3885890, gi|56784479, gi|12039318, gi|6319146 | 0.004351583 |
| cellular homeostasis | gi|21686526, gi|57899183, gi|46389828, gi|32487506, gi|51090743 | 0.01040998 |
| regulation of biological quality | gi|21686526, gi|57899183, gi|46389828, gi|32487506, gi|51090743, gi|34851127 | 0.01088926 |
| homeostatic process | gi|21686526, gi|57899183, gi|46389828, gi|32487506, gi|51090743 | 0.01254363 |
| positive regulation of molecular function | gi|57899183, gi|32487506 | 0.0133003 |
| biological regulation | gi|41052905, gi|32487506, gi|51090743, gi|21686526, gi|46389828, gi|57899183, gi|50878396, gi|29124123, gi|34851127 | 0.01447437 |
| chromatin organization | gi|3885890, gi|12039318, gi|6319146 | 0.01958553 |
| chromosome organization | gi|3885890, gi|12039318, gi|6319146 | 0.02254598 |
| phenylpropanoid biosynthetic process | gi|5257275, gi|50878396 | 0.03006896 |
| response to disaccharide stimulus | gi|50878396, gi|77552436 | 0.03006896 |
| response to abiotic stimulus | gi|3789954, gi|24431603, gi|113578236, gi|125590644, gi|57899183, gi|50878396, gi|34851127, gi|6319146, gi|3789952 | 0.03471245 |
| multicellular organismal development | gi|50252685, gi|125590644, gi|41052565, gi|27260946, gi|6319146, gi|34851127 | 0.03683836 |
| aromatic compound biosynthetic process | gi|5257275, gi|50878396 | 0.03684795 |
| phenylpropanoid metabolic process | gi|5257275, gi|50878396 | 0.04415367 |
| growth | gi|125590644, gi|34851127 | 0.04415367 |
| post-embryonic development | gi|125590644, gi|27260946, gi|6319146 | 0.04515178 |
