# Supplementary material for: Quantitative Proteomic Analysis of the Rice (Oryza sativa L.) Salt Response
Source: PLoS One. 2015 Mar 20;10(3):e0120978. doi: 10.1371/journal.pone.0120978 (PMC4368772; doi:10.1371/journal.pone.0120978)
Supplement: S5 Table — (DOC) [file pone.0120978.s005.doc]

**S5 Table. The information of spectrums for each differentially expressed proteins.**

| Accession | Description | Exp_mz^a^ | Exp_mr^b^ | Z^c^ | Calc_mr^d^ | Delta^e^ | Start^f^ | End^g^ | Miss^h^ | Before^i^ | Seq^j^ | After^k^ |
| --- | --- | --- | --- | --- | --- | --- | --- | --- | --- | --- | --- | --- |
| gi|29367391 | chloroplast photosystem I reaction center subunit II precursor-like protein (PsaD) | 978.9927 | 1955.971 | 2 | 1955.975 | -0.0044 | 102 | 116 | 0 | K | EQVFEMPTGGAAIMR | E |
|  |  | 970.9948 | 1939.975 | 2 | 1939.98 | -0.0052 | 102 | 116 | 0 | K | EQVFEMPTGGAAIMR | E |
|  |  | 802.7746 | 2405.302 | 3 | 2405.309 | -0.0072 | 87 | 101 | 0 | K | AQVEEFYVITWTSPK | E |
|  |  | 978.9926 | 1955.971 | 2 | 1955.975 | -0.0045 | 102 | 116 | 0 | K | EQVFEMPTGGAAIMR | E |
|  |  | 802.775 | 2405.303 | 3 | 2405.309 | -0.006 | 87 | 101 | 0 | K | AQVEEFYVITWTSPK | E |
|  |  | 986.9887 | 1971.963 | 2 | 1971.97 | -0.0072 | 102 | 116 | 0 | K | EQVFEMPTGGAAIMR | E |
|  |  | 970.9944 | 1939.974 | 2 | 1939.98 | -0.006 | 102 | 116 | 0 | K | EQVFEMPTGGAAIMR | E |
|  |  | 802.7744 | 2405.301 | 3 | 2405.309 | -0.008 | 87 | 101 | 0 | K | AQVEEFYVITWTSPK | E |
|  |  | 652.9976 | 1955.971 | 3 | 1955.975 | -0.0041 | 102 | 116 | 0 | K | EQVFEMPTGGAAIMR | E |
|  |  | 970.993 | 1939.971 | 2 | 1939.98 | -0.0088 | 102 | 116 | 0 | K | EQVFEMPTGGAAIMR | E |
|  |  | 978.9917 | 1955.969 | 2 | 1955.975 | -0.0063 | 102 | 116 | 0 | K | EQVFEMPTGGAAIMR | E |
|  |  | 802.7744 | 2405.301 | 3 | 2405.309 | -0.0078 | 87 | 101 | 0 | K | AQVEEFYVITWTSPK | E |
|  |  | 970.9923 | 1939.97 | 2 | 1939.98 | -0.0102 | 102 | 116 | 0 | K | EQVFEMPTGGAAIMR | E |
|  |  | 970.994 | 1939.974 | 2 | 1939.98 | -0.0068 | 102 | 116 | 0 | K | EQVFEMPTGGAAIMR | E |
|  |  | 970.9936 | 1939.973 | 2 | 1939.98 | -0.0076 | 102 | 116 | 0 | K | EQVFEMPTGGAAIMR | E |
|  |  | 978.9926 | 1955.971 | 2 | 1955.975 | -0.0046 | 102 | 116 | 0 | K | EQVFEMPTGGAAIMR | E |
|  |  | 802.7745 | 2405.302 | 3 | 2405.309 | -0.0074 | 87 | 101 | 0 | K | AQVEEFYVITWTSPK | E |
|  |  | 970.9949 | 1939.975 | 2 | 1939.98 | -0.0049 | 102 | 116 | 0 | K | EQVFEMPTGGAAIMR | E |
|  |  | 1203.66 | 2405.305 | 2 | 2405.309 | -0.0047 | 87 | 101 | 0 | K | AQVEEFYVITWTSPK | E |
|  |  | 978.9916 | 1955.969 | 2 | 1955.975 | -0.0064 | 102 | 116 | 0 | K | EQVFEMPTGGAAIMR | E |
|  |  | 802.7753 | 2405.304 | 3 | 2405.309 | -0.0052 | 87 | 101 | 0 | K | AQVEEFYVITWTSPK | E |
|  |  | 970.9939 | 1939.973 | 2 | 1939.98 | -0.007 | 102 | 116 | 0 | K | EQVFEMPTGGAAIMR | E |
|  |  | 647.6658 | 1939.976 | 3 | 1939.98 | -0.0046 | 102 | 116 | 0 | K | EQVFEMPTGGAAIMR | E |
|  |  | 970.9957 | 1939.977 | 2 | 1939.98 | -0.0033 | 102 | 116 | 0 | K | EQVFEMPTGGAAIMR | E |
|  |  | 802.775 | 2405.303 | 3 | 2405.309 | -0.006 | 87 | 101 | 0 | K | AQVEEFYVITWTSPK | E |
|  |  | 970.9943 | 1939.974 | 2 | 1939.98 | -0.0062 | 102 | 116 | 0 | K | EQVFEMPTGGAAIMR | E |
|  |  | 970.9935 | 1939.972 | 2 | 1939.98 | -0.0079 | 102 | 116 | 0 | K | EQVFEMPTGGAAIMR | E |
|  |  | 978.9923 | 1955.97 | 2 | 1955.975 | -0.0051 | 102 | 116 | 0 | K | EQVFEMPTGGAAIMR | E |
|  |  | 802.7754 | 2405.305 | 3 | 2405.309 | -0.0047 | 87 | 101 | 0 | K | AQVEEFYVITWTSPK | E |
|  |  | 1203.659 | 2405.303 | 2 | 2405.309 | -0.0062 | 87 | 101 | 0 | K | AQVEEFYVITWTSPK | E |
|  |  | 970.9939 | 1939.973 | 2 | 1939.98 | -0.007 | 102 | 116 | 0 | K | EQVFEMPTGGAAIMR | E |
| gi|3885894 | photosystem-1 H subunit GOS5 (PsaH) | 634.0184 | 1899.034 | 3 | 1899.039 | -0.0058 | 86 | 96 | 0 | K | FFETFAGPFTK | R |
|  |  | 634.0181 | 1899.033 | 3 | 1899.039 | -0.0067 | 86 | 96 | 0 | K | FFETFAGPFTK | R |
|  |  | 634.0194 | 1899.036 | 3 | 1899.039 | -0.0028 | 86 | 96 | 0 | K | FFETFAGPFTK | R |
|  |  | 634.0196 | 1899.037 | 3 | 1899.039 | -0.0021 | 86 | 96 | 0 | K | FFETFAGPFTK | R |
| gi|3789954 | chlorophyll a/b-binding protein precursor (Lhca1) | 982.5391 | 1963.064 | 2 | 1963.067 | -0.0029 | 164 | 176 | 0 | K | YPGGAFDPLGFSK | D |
|  |  | 655.3613 | 1963.062 | 3 | 1963.067 | -0.0045 | 164 | 176 | 0 | K | YPGGAFDPLGFSK | D |
|  |  | 982.5381 | 1963.062 | 2 | 1963.067 | -0.0049 | 164 | 176 | 0 | K | YPGGAFDPLGFSK | D |
|  |  | 982.5388 | 1963.063 | 2 | 1963.067 | -0.0034 | 164 | 176 | 0 | K | YPGGAFDPLGFSK | D |
|  |  | 655.3613 | 1963.062 | 3 | 1963.067 | -0.0043 | 164 | 176 | 0 | K | YPGGAFDPLGFSK | D |
| gi|34393511 | putative photosystem I antenna protein (Lhca2) | 1105.907 | 3314.699 | 3 | 3314.707 | -0.0082 | 182 | 207 | 0 | K | LTGTDVGYPGGLWFDPLGWGTGSPEK | I |
|  |  | 830.4735 | 2488.399 | 3 | 2488.401 | -0.0026 | 106 | 122 | 0 | R | WAMLGAAGIFIPEFLTK | I |
|  |  | 1105.907 | 3314.698 | 3 | 3314.707 | -0.0097 | 182 | 207 | 0 | K | LTGTDVGYPGGLWFDPLGWGTGSPEK | I |
|  |  | 830.4731 | 2488.397 | 3 | 2488.401 | -0.0039 | 106 | 122 | 0 | R | WAMLGAAGIFIPEFLTK | I |
| gi|3789952 | chlorophyll a/b-binding protein precursor (Lhca4) | 818.4755 | 2452.405 | 3 | 2452.405 | -0.0002 | 94 | 110 | 0 | R | WAMLGVAGMLLPEVLTK | I |
|  |  | 1078.101 | 2154.187 | 2 | 2154.194 | -0.0061 | 111 | 124 | 0 | K | IGLIDAPQWYDAGK | A |
|  |  | 813.1402 | 2436.399 | 3 | 2436.41 | -0.011 | 94 | 110 | 0 | R | WAMLGVAGMLLPEVLTK | I |
|  |  | 664.388 | 2653.523 | 4 | 2653.524 | -0.001 | 203 | 220 | 0 | R | LAMLAFLGFLVQHNVTQK | G |
|  |  | 664.3875 | 2653.521 | 4 | 2653.524 | -0.003 | 203 | 220 | 0 | R | LAMLAFLGFLVQHNVTQK | G |
|  |  | 813.1422 | 2436.405 | 3 | 2436.41 | -0.005 | 94 | 110 | 0 | R | WAMLGVAGMLLPEVLTK | I |
|  |  | 813.1418 | 2436.404 | 3 | 2436.41 | -0.0063 | 94 | 110 | 0 | R | WAMLGVAGMLLPEVLTK | I |
|  |  | 719.0701 | 2154.189 | 3 | 2154.194 | -0.0049 | 111 | 124 | 0 | K | IGLIDAPQWYDAGK | A |
|  |  | 818.4744 | 2452.402 | 3 | 2452.405 | -0.0032 | 94 | 110 | 0 | R | WAMLGVAGMLLPEVLTK | I |
|  |  | 818.4743 | 2452.401 | 3 | 2452.405 | -0.0036 | 94 | 110 | 0 | R | WAMLGVAGMLLPEVLTK | I |
|  |  | 813.1418 | 2436.404 | 3 | 2436.41 | -0.0063 | 94 | 110 | 0 | R | WAMLGVAGMLLPEVLTK | I |
|  |  | 1078.101 | 2154.188 | 2 | 2154.194 | -0.0056 | 111 | 124 | 0 | K | IGLIDAPQWYDAGK | A |
| gi|18855008 | putative chloroplast chaperonin | 901.4885 | 2701.444 | 3 | 2701.446 | -0.002 | 88 | 107 | 0 | R | YLMGEILSVGADVNEVEAGK | K |
|  |  | 739.4811 | 1476.948 | 2 | 1476.949 | -0.0013 | 72 | 80 | 0 | K | SVGGVLLPK | S |
|  |  | 901.4866 | 2701.438 | 3 | 2701.446 | -0.0079 | 88 | 107 | 0 | R | YLMGEILSVGADVNEVEAGK | K |
| gi|51090743 | putative peroxiredoxin Q | 669.3766 | 1336.739 | 2 | 1336.741 | -0.002 | 73 | 82 | 0 | K | GSAAPNFTLR | D |
|  |  | 960.0177 | 1918.021 | 2 | 1918.026 | -0.0047 | 168 | 182 | 0 | K | EWGVPADLFGTLPGR | Q |
|  |  | 640.3472 | 1918.02 | 3 | 1918.026 | -0.0057 | 168 | 182 | 0 | K | EWGVPADLFGTLPGR | Q |
|  |  | 439.2864 | 876.5583 | 2 | 876.5587 | -0.0004 | 213 | 217 | 0 | K | ILQSL | - |
|  |  | 640.3475 | 1918.021 | 3 | 1918.026 | -0.0048 | 168 | 182 | 0 | K | EWGVPADLFGTLPGR | Q |
|  |  | 784.4471 | 2350.319 | 3 | 2350.326 | -0.0065 | 167 | 182 | 1 | R | KEWGVPADLFGTLPGR | Q |
|  |  | 960.0189 | 1918.023 | 2 | 1918.026 | -0.0023 | 168 | 182 | 0 | K | EWGVPADLFGTLPGR | Q |
|  |  | 960.0168 | 1918.019 | 2 | 1918.026 | -0.0066 | 168 | 182 | 0 | K | EWGVPADLFGTLPGR | Q |
|  |  | 439.2865 | 876.5585 | 2 | 876.5587 | -0.0002 | 213 | 217 | 0 | K | ILQSL | - |
|  |  | 960.0168 | 1918.019 | 2 | 1918.026 | -0.0066 | 168 | 182 | 0 | K | EWGVPADLFGTLPGR | Q |
|  |  | 784.4468 | 2350.319 | 3 | 2350.326 | -0.0072 | 167 | 182 | 1 | R | KEWGVPADLFGTLPGR | Q |
|  |  | 669.3757 | 1336.737 | 2 | 1336.741 | -0.0037 | 73 | 82 | 0 | K | GSAAPNFTLR | D |
|  |  | 669.3766 | 1336.739 | 2 | 1336.741 | -0.002 | 73 | 82 | 0 | K | GSAAPNFTLR | D |
|  |  | 648.0402 | 1941.099 | 3 | 1941.103 | -0.0046 | 153 | 164 | 0 | K | LPFTLLSDEGNK | V |
|  |  | 960.0174 | 1918.02 | 2 | 1918.026 | -0.0053 | 168 | 182 | 0 | K | EWGVPADLFGTLPGR | Q |
|  |  | 640.3483 | 1918.023 | 3 | 1918.026 | -0.0026 | 168 | 182 | 0 | K | EWGVPADLFGTLPGR | Q |
|  |  | 960.0173 | 1918.02 | 2 | 1918.026 | -0.0056 | 168 | 182 | 0 | K | EWGVPADLFGTLPGR | Q |
|  |  | 784.4473 | 2350.32 | 3 | 2350.326 | -0.0059 | 167 | 182 | 1 | R | KEWGVPADLFGTLPGR | Q |
|  |  | 640.3474 | 1918.02 | 3 | 1918.026 | -0.0053 | 168 | 182 | 0 | K | EWGVPADLFGTLPGR | Q |
|  |  | 640.3477 | 1918.021 | 3 | 1918.026 | -0.0044 | 168 | 182 | 0 | K | EWGVPADLFGTLPGR | Q |
|  |  | 669.3755 | 1336.737 | 2 | 1336.741 | -0.004 | 73 | 82 | 0 | K | GSAAPNFTLR | D |
|  |  | 669.3756 | 1336.737 | 2 | 1336.741 | -0.0039 | 73 | 82 | 0 | K | GSAAPNFTLR | D |
|  |  | 960.0187 | 1918.023 | 2 | 1918.026 | -0.0026 | 168 | 182 | 0 | K | EWGVPADLFGTLPGR | Q |
|  |  | 784.4473 | 2350.32 | 3 | 2350.326 | -0.0057 | 167 | 182 | 1 | R | KEWGVPADLFGTLPGR | Q |
|  |  | 648.0403 | 1941.099 | 3 | 1941.103 | -0.0042 | 153 | 164 | 0 | K | LPFTLLSDEGNK | V |
|  |  | 960.0174 | 1918.02 | 2 | 1918.026 | -0.0053 | 168 | 182 | 0 | K | EWGVPADLFGTLPGR | Q |
|  |  | 669.3759 | 1336.737 | 2 | 1336.741 | -0.0034 | 73 | 82 | 0 | K | GSAAPNFTLR | D |
| gi|46389828 | putative thioredoxin peroxidase | 516.9827 | 1547.926 | 3 | 1547.929 | -0.0023 | 115 | 122 | 0 | K | HLPGFIEK | A |
|  |  | 624.848 | 2495.363 | 4 | 2495.37 | -0.0071 | 172 | 189 | 0 | R | ALGVEMDLSDKPMGLGVR | S |
|  |  | 624.848 | 2495.363 | 4 | 2495.37 | -0.0074 | 172 | 189 | 0 | R | ALGVEMDLSDKPMGLGVR | S |
|  |  | 886.5226 | 1771.031 | 2 | 1771.034 | -0.0035 | 193 | 203 | 0 | R | YALLADDGVVK | V |
|  |  | 917.4825 | 2749.426 | 3 | 2749.431 | -0.0049 | 204 | 223 | 0 | K | VLNLEEGGAFTTSSAEEMLK | A |
|  |  | 624.8493 | 2495.368 | 4 | 2495.37 | -0.002 | 172 | 189 | 0 | R | ALGVEMDLSDKPMGLGVR | S |
|  |  | 832.7956 | 2495.365 | 3 | 2495.37 | -0.0052 | 172 | 189 | 0 | R | ALGVEMDLSDKPMGLGVR | S |
|  |  | 516.9826 | 1547.926 | 3 | 1547.929 | -0.0025 | 115 | 122 | 0 | K | HLPGFIEK | A |
|  |  | 912.1505 | 2733.43 | 3 | 2733.436 | -0.0059 | 204 | 223 | 0 | K | VLNLEEGGAFTTSSAEEMLK | A |
|  |  | 820.7737 | 2459.299 | 3 | 2459.305 | -0.0051 | 69 | 85 | 0 | K | LPDATLSYFDPADGELK | T |
|  |  | 917.4816 | 2749.423 | 3 | 2749.431 | -0.0074 | 204 | 223 | 0 | K | VLNLEEGGAFTTSSAEEMLK | A |
|  |  | 832.7959 | 2495.366 | 3 | 2495.37 | -0.0042 | 172 | 189 | 0 | R | ALGVEMDLSDKPMGLGVR | S |
|  |  | 886.5225 | 1771.03 | 2 | 1771.034 | -0.0037 | 193 | 203 | 0 | R | YALLADDGVVK | V |
|  |  | 886.5229 | 1771.031 | 2 | 1771.034 | -0.0029 | 193 | 203 | 0 | R | YALLADDGVVK | V |
| gi|57899183 | thioredoxin M-like | 731.1034 | 2190.288 | 3 | 2190.291 | -0.003 | 82 | 94 | 0 | R | YQIEALPTFIIFK | N |
|  |  | 731.1023 | 2190.285 | 3 | 2190.291 | -0.0063 | 82 | 94 | 0 | R | YQIEALPTFIIFK | N |
| gi|32487506 | thioredoxin x | 620.0599 | 1857.158 | 3 | 1857.159 | -0.0011 | 137 | 147 | 0 | K | VYGLPSLILFK | D |
|  |  | 620.0585 | 1857.154 | 3 | 1857.159 | -0.0053 | 137 | 147 | 0 | K | VYGLPSLILFK | D |
|  |  | 620.0602 | 1857.159 | 3 | 1857.159 | -0.0002 | 137 | 147 | 0 | K | VYGLPSLILFK | D |
|  |  | 620.0587 | 1857.154 | 3 | 1857.159 | -0.0046 | 137 | 147 | 0 | K | VYGLPSLILFK | D |
| gi|11177845 | putative glutathione S-transferase OsGSTF3 | 672.0275 | 2013.061 | 3 | 2013.066 | -0.0056 | 133 | 145 | 0 | R | ATDMAVVEQNEAK | L |
|  |  | 729.8889 | 1457.763 | 2 | 1457.767 | -0.0036 | 61 | 70 | 0 | K | DSLTTVFESR | A |
|  |  | 672.0293 | 2013.066 | 3 | 2013.066 | -0.0001 | 133 | 145 | 0 | R | ATDMAVVEQNEAK | L |
|  |  | 729.8894 | 1457.764 | 2 | 1457.767 | -0.0026 | 61 | 70 | 0 | K | DSLTTVFESR | A |
|  |  | 729.8888 | 1457.763 | 2 | 1457.767 | -0.0038 | 61 | 70 | 0 | K | DSLTTVFESR | A |
|  |  | 700.3809 | 2098.121 | 3 | 2098.123 | -0.0021 | 27 | 39 | 0 | K | DVPFQVEPVDMSK | G |
|  |  | 729.8889 | 1457.763 | 2 | 1457.767 | -0.0036 | 61 | 70 | 0 | K | DSLTTVFESR | A |
|  |  | 729.8889 | 1457.763 | 2 | 1457.767 | -0.0037 | 61 | 70 | 0 | K | DSLTTVFESR | A |
| gi|3885882 | inorganic pyrophosphatase | 851.144 | 2550.41 | 3 | 2550.415 | -0.0051 | 181 | 198 | 0 | K | EVAVNEFLPAEDAINAIK | Y |
|  |  | 851.1435 | 2550.409 | 3 | 2550.415 | -0.0068 | 181 | 198 | 0 | K | EVAVNEFLPAEDAINAIK | Y |
|  |  | 851.1434 | 2550.408 | 3 | 2550.415 | -0.0072 | 181 | 198 | 0 | K | EVAVNEFLPAEDAINAIK | Y |
| gi|28564802 | putative vacuolar ATP synthase subunit H | 589.8815 | 1177.748 | 2 | 1177.749 | -0.0006 | 60 | 66 | 0 | R | VFLNILR | N |
|  |  | 589.8812 | 1177.748 | 2 | 1177.749 | -0.0011 | 60 | 66 | 0 | R | VFLNILR | N |
|  |  | 589.8813 | 1177.748 | 2 | 1177.749 | -0.0008 | 60 | 66 | 0 | R | VFLNILR | N |
| gi|51535416 | putative ATP synthase delta chain | 557.3231 | 1668.948 | 3 | 1668.948 | -0.0007 | 138 | 146 | 0 | R | FVDLTMAHK | G |
|  |  | 769.7598 | 2306.258 | 3 | 2306.262 | -0.0044 | 101 | 116 | 0 | K | AITEIFAEAGFSDVTK | N |
|  |  | 769.7595 | 2306.257 | 3 | 2306.262 | -0.0052 | 101 | 116 | 0 | K | AITEIFAEAGFSDVTK | N |
|  |  | 557.3234 | 1668.948 | 3 | 1668.948 | 0 | 138 | 146 | 0 | R | FVDLTMAHK | G |
|  |  | 812.9876 | 1623.961 | 2 | 1623.966 | -0.0051 | 169 | 177 | 0 | K | ETLQDILGK | N |
|  |  | 941.5209 | 2821.541 | 3 | 2821.548 | -0.0065 | 39 | 60 | 0 | K | VPEALYGGTGNYASALFLTAAK | A |
| gi|41052565 | putative ATP synthase | 884.1043 | 2649.291 | 3 | 2649.298 | -0.0072 | 131 | 149 | 0 | K | SGLIDDMGAEAMMMEALEK | V |
|  |  | 837.4512 | 1672.888 | 2 | 1672.894 | -0.0061 | 70 | 82 | 0 | K | ITIDPDDPAAVSR | Y |
|  |  | 483.2968 | 1446.869 | 3 | 1446.87 | -0.001 | 49 | 55 | 0 | K | GIFFEVK | K |
| gi|108864431 | Acyl carrier protein 2, chloroplast precursor | 565.6674 | 1693.98 | 3 | 1693.982 | -0.0021 | 74 | 83 | 0 | K | QQLALGEEAK | L |
|  |  | 847.9976 | 1693.981 | 2 | 1693.982 | -0.0018 | 74 | 83 | 0 | K | QQLALGEEAK | L |
|  |  | 847.9969 | 1693.979 | 2 | 1693.982 | -0.0031 | 74 | 83 | 0 | K | QQLALGEEAK | L |
| gi|77553225 | Carboxyvinyl-carboxyphosphonate phosphorylmutase, putative, expressed | 811.0272 | 1620.04 | 2 | 1620.044 | -0.0036 | 268 | 277 | 0 | R | ALVDVLAALK | R |
|  |  | 541.0214 | 1620.042 | 3 | 1620.044 | -0.0013 | 268 | 277 | 0 | R | ALVDVLAALK | R |
|  |  | 727.4177 | 1452.821 | 2 | 1452.824 | -0.0035 | 257 | 267 | 0 | K | SPLTTVYAAAR | A |
|  |  | 727.4178 | 1452.821 | 2 | 1452.824 | -0.0033 | 257 | 267 | 0 | K | SPLTTVYAAAR | A |
|  |  | 541.0212 | 1620.042 | 3 | 1620.044 | -0.0018 | 268 | 277 | 0 | R | ALVDVLAALK | R |
|  |  | 811.0267 | 1620.039 | 2 | 1620.044 | -0.0047 | 268 | 277 | 0 | R | ALVDVLAALK | R |
| gi|24431603 | Putative transcription factor | 956.0421 | 1910.07 | 2 | 1910.072 | -0.0026 | 502 | 512 | 0 | K | DDVVIQFQNPK | V |
|  |  | 956.0421 | 1910.07 | 2 | 1910.072 | -0.0027 | 502 | 512 | 0 | K | DDVVIQFQNPK | V |
|  |  | 956.0417 | 1910.069 | 2 | 1910.072 | -0.0033 | 502 | 512 | 0 | K | DDVVIQFQNPK | V |
|  |  | 851.4792 | 2551.416 | 3 | 2551.422 | -0.0062 | 513 | 531 | 0 | K | VQASIGANTWVVSGTPQTK | K |
|  |  | 956.0426 | 1910.071 | 2 | 1910.072 | -0.0016 | 502 | 512 | 0 | K | DDVVIQFQNPK | V |
| gi|55296302 | putative MAR binding filament-like protein 1 | 725.3946 | 1448.775 | 2 | 1448.778 | -0.003 | 475 | 484 | 0 | K | LSVELDEANR | M |
|  |  | 725.3943 | 1448.774 | 2 | 1448.778 | -0.0037 | 475 | 484 | 0 | K | LSVELDEANR | M |
|  |  | 784.0712 | 2349.192 | 3 | 2349.197 | -0.0047 | 535 | 553 | 0 | K | GQEELEATSNELASIVEAR | D |
|  |  | 782.7304 | 2345.169 | 3 | 2345.177 | -0.0071 | 657 | 674 | 0 | K | ITTEAHENTEDAQNLISR | L |
|  |  | 935.4681 | 1868.922 | 2 | 1868.927 | -0.0053 | 456 | 469 | 0 | R | ESLQSTEEALTDSR | S |
|  |  | 784.0714 | 2349.192 | 3 | 2349.197 | -0.0044 | 535 | 553 | 0 | K | GQEELEATSNELASIVEAR | D |
|  |  | 935.4677 | 1868.921 | 2 | 1868.927 | -0.0061 | 456 | 469 | 0 | R | ESLQSTEEALTDSR | S |
|  |  | 935.468 | 1868.921 | 2 | 1868.927 | -0.0056 | 456 | 469 | 0 | R | ESLQSTEEALTDSR | S |
|  |  | 782.7305 | 2345.17 | 3 | 2345.177 | -0.0068 | 657 | 674 | 0 | K | ITTEAHENTEDAQNLISR | L |
|  |  | 784.0715 | 2349.193 | 3 | 2349.197 | -0.004 | 535 | 553 | 0 | K | GQEELEATSNELASIVEAR | D |
|  |  | 935.4674 | 1868.92 | 2 | 1868.927 | -0.0067 | 456 | 469 | 0 | R | ESLQSTEEALTDSR | S |
| gi|77552436 | auxin-repressed protein-like protein ARP1, putative, expressed | 583.6348 | 1747.883 | 3 | 1747.884 | -0.0011 | 36 | 47 | 0 | K | DGDGEASGAAYK | R |
|  |  | 583.6347 | 1747.882 | 3 | 1747.884 | -0.0015 | 36 | 47 | 0 | K | DGDGEASGAAYK | R |
| gi|33358444 | hydroperoxide lyase | 679.3461 | 2035.017 | 3 | 2035.02 | -0.0033 | 151 | 166 | 0 | R | AAVDDMLAAVEEDLNR | A |
|  |  | 679.346 | 2035.016 | 3 | 2035.02 | -0.0038 | 151 | 166 | 0 | R | AAVDDMLAAVEEDLNR | A |
|  |  | 679.3461 | 2035.016 | 3 | 2035.02 | -0.0035 | 151 | 166 | 0 | R | AAVDDMLAAVEEDLNR | A |
|  |  | 679.3461 | 2035.016 | 3 | 2035.02 | -0.0035 | 151 | 166 | 0 | R | AAVDDMLAAVEEDLNR | A |
|  |  | 679.3455 | 2035.015 | 3 | 2035.02 | -0.0053 | 151 | 166 | 0 | R | AAVDDMLAAVEEDLNR | A |
| gi|34015153 | putative CBS domain containing protein | 712.3691 | 1422.724 | 2 | 1422.726 | -0.0021 | 138 | 147 | 0 | R | ETTNLEDAAR | L |
|  |  | 707.6964 | 2120.067 | 3 | 2120.073 | -0.0052 | 46 | 61 | 0 | K | STTSVDEALEMLVEHR | I |
|  |  | 707.6964 | 2120.067 | 3 | 2120.073 | -0.0054 | 46 | 61 | 0 | K | STTSVDEALEMLVEHR | I |
| gi|28209481 | expressed protein | 744.3741 | 1486.734 | 2 | 1486.736 | -0.0023 | 13 | 23 | 0 | R | GFVADDDAFAR | S |
|  |  | 744.3737 | 1486.733 | 2 | 1486.736 | -0.003 | 13 | 23 | 0 | R | GFVADDDAFAR | S |
|  |  | 518.6288 | 1552.865 | 3 | 1552.867 | -0.0025 | 148 | 158 | 0 | R | AADAAAAADAK | - |
|  |  | 777.439 | 1552.864 | 2 | 1552.867 | -0.0035 | 148 | 158 | 0 | R | AADAAAAADAK | - |
| gi|5922611 | putative small GTP-binding protein Bsar1a | 905.478 | 1808.942 | 2 | 1808.946 | -0.0047 | 131 | 143 | 0 | K | IDIPYAASEEELR | Y |
|  |  | 905.4788 | 1808.943 | 2 | 1808.946 | -0.0032 | 131 | 143 | 0 | K | IDIPYAASEEELR | Y |
|  |  | 905.4784 | 1808.942 | 2 | 1808.946 | -0.0039 | 131 | 143 | 0 | K | IDIPYAASEEELR | Y |
|  |  | 905.4777 | 1808.941 | 2 | 1808.946 | -0.0054 | 131 | 143 | 0 | K | IDIPYAASEEELR | Y |
| gi|50878396 | putative P-II nitrogen sensing protein | 788.4911 | 1574.968 | 2 | 1574.97 | -0.0026 | 174 | 184 | 0 | K | IFLIPVSDVIR | I |
|  |  | 788.4918 | 1574.969 | 2 | 1574.97 | -0.0012 | 174 | 184 | 0 | K | IFLIPVSDVIR | I |
|  |  | 788.4907 | 1574.967 | 2 | 1574.97 | -0.0034 | 174 | 184 | 0 | K | IFLIPVSDVIR | I |
|  |  | 788.4922 | 1574.97 | 2 | 1574.97 | -0.0004 | 174 | 184 | 0 | K | IFLIPVSDVIR | I |
| gi|41052905 | putative small nuclear ribonucleoprotein polypeptide D3 | 572.6725 | 1714.996 | 3 | 1714.998 | -0.0019 | 72 | 80 | 0 | R | FMIIPDMLK | N |
|  |  | 572.6718 | 1714.994 | 3 | 1714.998 | -0.0039 | 72 | 80 | 0 | R | FMIIPDMLK | N |
|  |  | 572.6725 | 1714.996 | 3 | 1714.998 | -0.0019 | 72 | 80 | 0 | R | FMIIPDMLK | N |
|  |  | 572.6729 | 1714.997 | 3 | 1714.998 | -0.0008 | 72 | 80 | 0 | R | FMIIPDMLK | N |
| gi|113578236 | Os05g0154800 | 509.6638 | 1525.97 | 3 | 1525.969 | 0.0002 | 53 | 60 | 0 | K | ILDVLAFK | T |
|  |  | 509.6641 | 1525.97 | 3 | 1525.969 | 0.001 | 53 | 60 | 0 | K | ILDVLAFK | T |
| gi|50725625 | putative acidic ribosomal protein P1a | 945.0402 | 1888.066 | 2 | 1888.071 | -0.0049 | 38 | 48 | 0 | K | VEAYWPGLFAK | L |
|  |  | 945.0407 | 1888.067 | 2 | 1888.071 | -0.0039 | 38 | 48 | 0 | K | VEAYWPGLFAK | L |
|  |  | 945.0403 | 1888.066 | 2 | 1888.071 | -0.0047 | 38 | 48 | 0 | K | VEAYWPGLFAK | L |
|  |  | 630.3628 | 1888.067 | 3 | 1888.071 | -0.0043 | 38 | 48 | 0 | K | VEAYWPGLFAK | L |
| gi|25553579 | putative ribosomal protein S18 | 649.6979 | 1946.072 | 3 | 1946.076 | -0.0037 | 95 | 106 | 0 | R | FSQVVSNALDMK | L |
|  |  | 544.6715 | 1630.993 | 3 | 1630.994 | -0.0016 | 26 | 34 | 0 | K | IMFALTSIK | G |
|  |  | 774.4119 | 1546.809 | 2 | 1546.815 | -0.0052 | 56 | 66 | 0 | R | AGELTPEELER | L |
|  |  | 774.4119 | 1546.809 | 2 | 1546.815 | -0.0053 | 56 | 66 | 0 | R | AGELTPEELER | L |
|  |  | 649.6981 | 1946.073 | 3 | 1946.076 | -0.0031 | 95 | 106 | 0 | R | FSQVVSNALDMK | L |
|  |  | 774.4128 | 1546.811 | 2 | 1546.815 | -0.0035 | 56 | 66 | 0 | R | AGELTPEELER | L |
|  |  | 649.6981 | 1946.072 | 3 | 1946.076 | -0.0033 | 95 | 106 | 0 | R | FSQVVSNALDMK | L |
|  |  | 774.4122 | 1546.81 | 2 | 1546.815 | -0.0047 | 56 | 66 | 0 | R | AGELTPEELER | L |
|  |  | 544.6715 | 1630.993 | 3 | 1630.994 | -0.0014 | 26 | 34 | 0 | K | IMFALTSIK | G |
| gi|50252685 | putative ribosomal protein L10a | 754.4025 | 1506.791 | 2 | 1506.794 | -0.0039 | 11 | 21 | 0 | R | EAISQIANDSR | E |
|  |  | 754.4025 | 1506.791 | 2 | 1506.794 | -0.0039 | 11 | 21 | 0 | R | EAISQIANDSR | E |
| gi|11974 | ribosomal protein S2 | 711.7288 | 2132.165 | 3 | 2132.166 | -0.0017 | 12 | 25 | 0 | K | EMIEAGVHFGHGIK | K |
|  |  | 711.7284 | 2132.164 | 3 | 2132.166 | -0.0027 | 12 | 25 | 0 | K | EMIEAGVHFGHGIK | K |
|  |  | 681.4047 | 1360.795 | 2 | 1360.798 | -0.0032 | 80 | 90 | 0 | R | AADLVASAAIR | A |
| gi|37805854 | putative ribosomal protein L34 | 524.6462 | 1570.917 | 3 | 1570.918 | -0.0011 | 94 | 101 | 0 | R | AFLVEEQK | I |
|  |  | 524.6462 | 1570.917 | 3 | 1570.918 | -0.0013 | 94 | 101 | 0 | R | AFLVEEQK | I |
|  |  | 786.4648 | 1570.915 | 2 | 1570.918 | -0.0029 | 94 | 101 | 0 | R | AFLVEEQK | I |
|  |  | 786.4648 | 1570.915 | 2 | 1570.918 | -0.003 | 94 | 101 | 0 | R | AFLVEEQK | I |
| gi|14495192 | putative 26S proteasome subunit RPN9b | 673.7259 | 2018.156 | 3 | 2018.158 | -0.0025 | 310 | 321 | 0 | K | LSISDVEYLLMK | S |
|  |  | 673.7254 | 2018.154 | 3 | 2018.158 | -0.0039 | 310 | 321 | 0 | K | LSISDVEYLLMK | S |
| gi|11094192 | 26S proteasome regulatory particle triple-A ATPase subunit4 | 1053.084 | 2104.154 | 2 | 2104.155 | -0.0003 | 154 | 168 | 0 | R | ESIELPLMNPELFLR | V |
|  |  | 1053.084 | 2104.153 | 2 | 2104.155 | -0.0013 | 154 | 168 | 0 | R | ESIELPLMNPELFLR | V |
|  |  | 589.692 | 1766.054 | 3 | 1766.055 | -0.001 | 176 | 187 | 0 | K | GVLLYGPPGTGK | T |
|  |  | 884.0341 | 1766.054 | 2 | 1766.055 | -0.0016 | 176 | 187 | 0 | K | GVLLYGPPGTGK | T |
|  |  | 1053.083 | 2104.152 | 2 | 2104.155 | -0.0023 | 154 | 168 | 0 | R | ESIELPLMNPELFLR | V |
|  |  | 884.0338 | 1766.053 | 2 | 1766.055 | -0.0021 | 176 | 187 | 0 | K | GVLLYGPPGTGK | T |
|  |  | 702.3915 | 2104.153 | 3 | 2104.155 | -0.0017 | 154 | 168 | 0 | R | ESIELPLMNPELFLR | V |
| gi|12039318 | histone H4 | 640.3754 | 1918.104 | 3 | 1918.106 | -0.0016 | 81 | 92 | 0 | K | TVTAMDVVYALK | R |
|  |  | 960.0593 | 1918.104 | 2 | 1918.106 | -0.0019 | 81 | 92 | 0 | K | TVTAMDVVYALK | R |
|  |  | 960.0582 | 1918.102 | 2 | 1918.106 | -0.0041 | 81 | 92 | 0 | K | TVTAMDVVYALK | R |
|  |  | 967.5844 | 1933.154 | 2 | 1933.157 | -0.0027 | 25 | 36 | 0 | R | DNIQGITKPAIR | R |
|  |  | 960.0599 | 1918.105 | 2 | 1918.106 | -0.0006 | 81 | 92 | 0 | K | TVTAMDVVYALK | R |
|  |  | 640.3753 | 1918.104 | 3 | 1918.106 | -0.0018 | 81 | 92 | 0 | K | TVTAMDVVYALK | R |
|  |  | 960.0585 | 1918.103 | 2 | 1918.106 | -0.0034 | 81 | 92 | 0 | K | TVTAMDVVYALK | R |
|  |  | 640.3746 | 1918.102 | 3 | 1918.106 | -0.0038 | 81 | 92 | 0 | K | TVTAMDVVYALK | R |
|  |  | 742.9149 | 1483.815 | 2 | 1483.819 | -0.0036 | 47 | 56 | 0 | R | ISGLIYEETR | G |
|  |  | 640.3748 | 1918.103 | 3 | 1918.106 | -0.0034 | 81 | 92 | 0 | K | TVTAMDVVYALK | R |
|  |  | 967.5846 | 1933.155 | 2 | 1933.157 | -0.0024 | 25 | 36 | 0 | R | DNIQGITKPAIR | R |
|  |  | 960.058 | 1918.101 | 2 | 1918.106 | -0.0045 | 81 | 92 | 0 | K | TVTAMDVVYALK | R |
|  |  | 489.5889 | 1465.745 | 3 | 1465.747 | -0.0018 | 69 | 78 | 0 | R | DAVTYTEHAR | R |
|  |  | 640.3747 | 1918.102 | 3 | 1918.106 | -0.0037 | 81 | 92 | 0 | K | TVTAMDVVYALK | R |
| gi|6319146 | H2A protein | 870.9945 | 1739.974 | 2 | 1739.977 | -0.0023 | 107 | 116 | 0 | R | GDEELDTLIK | G |
|  |  | 870.9937 | 1739.973 | 2 | 1739.977 | -0.0039 | 107 | 116 | 0 | R | GDEELDTLIK | G |
|  |  | 870.994 | 1739.973 | 2 | 1739.977 | -0.0033 | 107 | 116 | 0 | R | GDEELDTLIK | G |
| gi|3885890 | histone H3 | 568.3516 | 1134.689 | 2 | 1134.692 | -0.0029 | 58 | 64 | 0 | K | STELLIR | K |
|  |  | 568.352 | 1134.689 | 2 | 1134.692 | -0.0021 | 58 | 64 | 0 | K | STELLIR | K |
|  |  | 568.3519 | 1134.689 | 2 | 1134.692 | -0.0023 | 58 | 64 | 0 | K | STELLIR | K |
|  |  | 568.3516 | 1134.689 | 2 | 1134.692 | -0.0028 | 58 | 64 | 0 | K | STELLIR | K |
| gi|29124123 | putative actin depolymerizing factor | 833.7789 | 2498.315 | 3 | 2498.32 | -0.0047 | 67 | 82 | 0 | R | FAIYDFDFLTAEDVPK | S |
|  |  | 687.8513 | 1373.688 | 2 | 1373.692 | -0.0035 | 101 | 109 | 0 | K | MLYASSNER | F |
|  |  | 833.7789 | 2498.315 | 3 | 2498.32 | -0.0045 | 67 | 82 | 0 | R | FAIYDFDFLTAEDVPK | S |
|  |  | 833.779 | 2498.315 | 3 | 2498.32 | -0.0043 | 67 | 82 | 0 | R | FAIYDFDFLTAEDVPK | S |
|  |  | 833.7784 | 2498.313 | 3 | 2498.32 | -0.0061 | 67 | 82 | 0 | R | FAIYDFDFLTAEDVPK | S |
| gi|34851127 | actin | 684.6997 | 2051.077 | 3 | 2051.084 | -0.007 | 241 | 256 | 0 | K | SYELPDGQVITIGAER | F |
|  |  | 765.9356 | 1529.857 | 2 | 1529.858 | -0.0018 | 186 | 193 | 0 | R | DLTDSLMK | I |
|  |  | 586.0088 | 1755.005 | 3 | 1755.006 | -0.0016 | 318 | 328 | 0 | K | EITALAPSSMK | I |
|  |  | 640.8285 | 1279.643 | 2 | 1279.646 | -0.0038 | 21 | 30 | 0 | K | AGFAGDDAPR | A |
|  |  | 597.6498 | 1789.928 | 3 | 1789.931 | -0.0032 | 53 | 63 | 0 | K | DAYVGDEAQSK | R |
|  |  | 1026.546 | 2051.078 | 2 | 2051.084 | -0.0059 | 241 | 256 | 0 | K | SYELPDGQVITIGAER | F |
|  |  | 878.5086 | 1755.003 | 2 | 1755.006 | -0.0035 | 318 | 328 | 0 | K | EITALAPSSMK | I |
|  |  | 578.6289 | 1732.865 | 3 | 1732.869 | -0.004 | 362 | 374 | 0 | K | GEYDESGPAIVHR | K |
|  |  | 1281.737 | 2561.459 | 2 | 2561.468 | -0.0092 | 98 | 115 | 0 | R | VAPEEHPVLLTEAPLNPK | A |
|  |  | 455.7437 | 1818.946 | 4 | 1818.947 | -0.0014 | 87 | 97 | 0 | K | IWHHTFYNELR | V |
|  |  | 684.7006 | 2051.08 | 3 | 2051.084 | -0.0043 | 241 | 256 | 0 | K | SYELPDGQVITIGAER | F |
|  |  | 1026.545 | 2051.075 | 2 | 2051.084 | -0.0094 | 241 | 256 | 0 | K | SYELPDGQVITIGAER | F |
|  |  | 878.5087 | 1755.003 | 2 | 1755.006 | -0.0034 | 318 | 328 | 0 | K | EITALAPSSMK | I |
|  |  | 640.8285 | 1279.642 | 2 | 1279.646 | -0.0039 | 21 | 30 | 0 | K | AGFAGDDAPR | A |
|  |  | 1026.547 | 2051.079 | 2 | 2051.084 | -0.0055 | 241 | 256 | 0 | K | SYELPDGQVITIGAER | F |
|  |  | 640.8267 | 1279.639 | 2 | 1279.646 | -0.0076 | 21 | 30 | 0 | K | AGFAGDDAPR | A |
|  |  | 584.9944 | 1751.961 | 3 | 1751.964 | -0.0023 | 42 | 52 | 0 | R | HTGVMVGMGQK | D |
|  |  | 591.3406 | 1771 | 3 | 1771.001 | -0.001 | 318 | 328 | 0 | K | EITALAPSSMK | I |
|  |  | 578.629 | 1732.865 | 3 | 1732.869 | -0.0034 | 362 | 374 | 0 | K | GEYDESGPAIVHR | K |
|  |  | 597.6497 | 1789.927 | 3 | 1789.931 | -0.0034 | 53 | 63 | 0 | K | DAYVGDEAQSK | R |
|  |  | 586.0085 | 1755.004 | 3 | 1755.006 | -0.0025 | 318 | 328 | 0 | K | EITALAPSSMK | I |
|  |  | 584.9943 | 1751.961 | 3 | 1751.964 | -0.0027 | 42 | 52 | 0 | R | HTGVMVGMGQK | D |
|  |  | 1026.545 | 2051.076 | 2 | 2051.084 | -0.0079 | 241 | 256 | 0 | K | SYELPDGQVITIGAER | F |
|  |  | 1026.546 | 2051.078 | 2 | 2051.084 | -0.0064 | 241 | 256 | 0 | K | SYELPDGQVITIGAER | F |
|  |  | 640.8289 | 1279.643 | 2 | 1279.646 | -0.0031 | 21 | 30 | 0 | K | AGFAGDDAPR | A |
|  |  | 718.8678 | 1435.721 | 2 | 1435.725 | -0.004 | 199 | 208 | 0 | R | GYSFTTTAER | E |
|  |  | 640.8287 | 1279.643 | 2 | 1279.646 | -0.0034 | 21 | 30 | 0 | K | AGFAGDDAPR | A |
|  |  | 876.988 | 1751.961 | 2 | 1751.964 | -0.0022 | 42 | 52 | 0 | R | HTGVMVGMGQK | D |
|  |  | 765.9358 | 1529.857 | 2 | 1529.858 | -0.0014 | 186 | 193 | 0 | R | DLTDSLMK | I |
|  |  | 1026.546 | 2051.078 | 2 | 2051.084 | -0.0059 | 241 | 256 | 0 | K | SYELPDGQVITIGAER | F |
|  |  | 765.9359 | 1529.857 | 2 | 1529.858 | -0.0012 | 186 | 193 | 0 | R | DLTDSLMK | I |
|  |  | 640.8267 | 1279.639 | 2 | 1279.646 | -0.0075 | 21 | 30 | 0 | K | AGFAGDDAPR | A |
|  |  | 765.9352 | 1529.856 | 2 | 1529.858 | -0.0026 | 186 | 193 | 0 | R | DLTDSLMK | I |
|  |  | 684.7005 | 2051.08 | 3 | 2051.084 | -0.0045 | 241 | 256 | 0 | K | SYELPDGQVITIGAER | F |
|  |  | 1026.545 | 2051.076 | 2 | 2051.084 | -0.0086 | 241 | 256 | 0 | K | SYELPDGQVITIGAER | F |
|  |  | 878.5085 | 1755.003 | 2 | 1755.006 | -0.0036 | 318 | 328 | 0 | K | EITALAPSSMK | I |
|  |  | 1281.739 | 2561.463 | 2 | 2561.468 | -0.0053 | 98 | 115 | 0 | R | VAPEEHPVLLTEAPLNPK | A |
|  |  | 718.8655 | 1435.716 | 2 | 1435.725 | -0.0086 | 199 | 208 | 0 | R | GYSFTTTAER | E |
|  |  | 886.5063 | 1770.998 | 2 | 1771.001 | -0.003 | 318 | 328 | 0 | K | EITALAPSSMK | I |
|  |  | 895.9706 | 1789.927 | 2 | 1789.931 | -0.0041 | 53 | 63 | 0 | K | DAYVGDEAQSK | R |
|  |  | 718.8673 | 1435.72 | 2 | 1435.725 | -0.0051 | 199 | 208 | 0 | R | GYSFTTTAER | E |
|  |  | 597.6495 | 1789.927 | 3 | 1789.931 | -0.004 | 53 | 63 | 0 | K | DAYVGDEAQSK | R |
|  |  | 684.7002 | 2051.079 | 3 | 2051.084 | -0.0054 | 241 | 256 | 0 | K | SYELPDGQVITIGAER | F |
|  |  | 1026.547 | 2051.079 | 2 | 2051.084 | -0.0052 | 241 | 256 | 0 | K | SYELPDGQVITIGAER | F |
|  |  | 878.5093 | 1755.004 | 2 | 1755.006 | -0.0021 | 318 | 328 | 0 | K | EITALAPSSMK | I |
|  |  | 847.7673 | 2540.28 | 3 | 2540.283 | -0.0031 | 71 | 86 | 0 | K | YPIEHGIVSNWDDMEK | I |
|  |  | 854.8284 | 2561.464 | 3 | 2561.468 | -0.0044 | 98 | 115 | 0 | R | VAPEEHPVLLTEAPLNPK | A |
|  |  | 597.6504 | 1789.929 | 3 | 1789.931 | -0.0014 | 53 | 63 | 0 | K | DAYVGDEAQSK | R |
|  |  | 847.767 | 2540.279 | 3 | 2540.283 | -0.004 | 71 | 86 | 0 | K | YPIEHGIVSNWDDMEK | I |
| gi|27260946 | putative isopentenyl pyrophosphate:dimethyllallyl pyrophosphate isomerase | 927.4973 | 1852.98 | 2 | 1852.984 | -0.0036 | 99 | 111 | 0 | R | ESELIQENYLGVR | N |
|  |  | 927.4965 | 1852.978 | 2 | 1852.984 | -0.0053 | 99 | 111 | 0 | R | ESELIQENYLGVR | N |
|  |  | 927.4973 | 1852.98 | 2 | 1852.984 | -0.0038 | 99 | 111 | 0 | R | ESELIQENYLGVR | N |
|  |  | 722.7268 | 2165.158 | 3 | 2165.161 | -0.0027 | 223 | 236 | 0 | K | GTLNEAVDMETIHK | L |
|  |  | 722.7259 | 2165.156 | 3 | 2165.161 | -0.0053 | 223 | 236 | 0 | K | GTLNEAVDMETIHK | L |
|  |  | 927.497 | 1852.98 | 2 | 1852.984 | -0.0042 | 99 | 111 | 0 | R | ESELIQENYLGVR | N |
| gi|34393921 | putative isocitrate lyase | 643.7012 | 1928.082 | 3 | 1928.086 | -0.0045 | 283 | 296 | 0 | R | SLAAVLSDAMSAGK | N |
|  |  | 638.3502 | 1912.029 | 3 | 1912.036 | -0.0076 | 323 | 335 | 0 | R | DAIASLNATDADK | Q |
| gi|5257275 | putative caffeoyl-CoA O-methyltransferase 1 | 740.9358 | 1479.857 | 2 | 1479.86 | -0.0032 | 34 | 43 | 0 | K | YVLDTTVLPR | E |
|  |  | 740.9361 | 1479.858 | 2 | 1479.86 | -0.0027 | 34 | 43 | 0 | K | YVLDTTVLPR | E |
| gi|21686526 | ferritin | 739.4185 | 2215.234 | 3 | 2215.238 | -0.0046 | 169 | 183 | 0 | K | GDALYAMELALALEK | L |
|  |  | 777.1118 | 2328.314 | 3 | 2328.319 | -0.0054 | 50 | 64 | 0 | K | EVLSGVVFQPFEELK | G |
|  |  | 777.1128 | 2328.317 | 3 | 2328.319 | -0.0025 | 50 | 64 | 0 | K | EVLSGVVFQPFEELK | G |
|  |  | 739.4191 | 2215.235 | 3 | 2215.238 | -0.0029 | 169 | 183 | 0 | K | GDALYAMELALALEK | L |
| gi|125600465 | hypothetical protein OsJ_24479 | 792.8976 | 1583.781 | 2 | 1583.785 | -0.0039 | 65 | 77 | 0 | K | DAEGAGIYGSQGR | D |
|  |  | 792.8974 | 1583.78 | 2 | 1583.785 | -0.0044 | 65 | 77 | 0 | K | DAEGAGIYGSQGR | D |
|  |  | 792.8976 | 1583.781 | 2 | 1583.785 | -0.0039 | 65 | 77 | 0 | K | DAEGAGIYGSQGR | D |
|  |  | 792.8979 | 1583.781 | 2 | 1583.785 | -0.0034 | 65 | 77 | 0 | K | DAEGAGIYGSQGR | D |
|  |  | 528.9343 | 1583.781 | 3 | 1583.785 | -0.0035 | 65 | 77 | 0 | K | DAEGAGIYGSQGR | D |
| gi|62701927 | CBS domain, putative | 645.013 | 1932.017 | 3 | 1932.022 | -0.0047 | 179 | 195 | 0 | K | AAEQGSALAAAVEGVER | Q |
|  |  | 645.0127 | 1932.016 | 3 | 1932.022 | -0.0056 | 179 | 195 | 0 | K | AAEQGSALAAAVEGVER | Q |
| gi|50252988 | unknown protein | 617.3971 | 1849.169 | 3 | 1849.169 | 0.0004 | 137 | 147 | 0 | K | FLGLPLPPFLK | I |
|  |  | 617.397 | 1849.169 | 3 | 1849.169 | 0 | 137 | 147 | 0 | K | FLGLPLPPFLK | I |
|  |  | 617.3968 | 1849.169 | 3 | 1849.169 | -0.0004 | 137 | 147 | 0 | K | FLGLPLPPFLK | I |
|  |  | 617.397 | 1849.169 | 3 | 1849.169 | 0 | 137 | 147 | 0 | K | FLGLPLPPFLK | I |
| gi|125590644 | hypothetical protein OsJ_15076 | 842.4543 | 2524.341 | 3 | 2524.344 | -0.0027 | 17 | 40 | 0 | K | AAAGLGAAASLLSASLYTVDGGER | A |
|  |  | 842.4543 | 2524.341 | 3 | 2524.344 | -0.0029 | 17 | 40 | 0 | K | AAAGLGAAASLLSASLYTVDGGER | A |
|  |  | 753.933 | 1505.852 | 2 | 1505.854 | -0.0027 | 89 | 98 | 0 | K | DLQMVNLTLR | L |
|  |  | 842.4552 | 2524.344 | 3 | 2524.344 | -0.0002 | 17 | 40 | 0 | K | AAAGLGAAASLLSASLYTVDGGER | A |
| gi|53749372 | unknown protein | 708.6752 | 2123.004 | 3 | 2123.007 | -0.0037 | 231 | 249 | 0 | R | YPDDGAAAGGGEAVGDELR | T |
|  |  | 708.6754 | 2123.004 | 3 | 2123.007 | -0.003 | 231 | 249 | 0 | R | YPDDGAAAGGGEAVGDELR | T |
|  |  | 708.6752 | 2123.004 | 3 | 2123.007 | -0.0037 | 231 | 249 | 0 | R | YPDDGAAAGGGEAVGDELR | T |
| gi|56784479 | hypothetical protein | 583.8606 | 2331.413 | 4 | 2331.414 | -0.0006 | 409 | 423 | 0 | K | AELQFLAILPDIRPK | V |
|  |  | 580.682 | 1739.024 | 3 | 1739.027 | -0.0023 | 395 | 404 | 0 | K | GLVVWVMEAK | V |
|  |  | 583.8602 | 2331.412 | 4 | 2331.414 | -0.0023 | 409 | 423 | 0 | K | AELQFLAILPDIRPK | V |
|  |  | 580.682 | 1739.024 | 3 | 1739.027 | -0.0023 | 395 | 404 | 0 | K | GLVVWVMEAK | V |
| gi|62701926 | abscisic acid- and stress-induced protein - rice | 1102.584 | 2203.153 | 2 | 2203.158 | -0.0053 | 62 | 80 | 0 | K | QHLGEAGALAAGAFALYEK | H |
|  |  | 735.3912 | 2203.152 | 3 | 2203.158 | -0.0061 | 62 | 80 | 0 | K | QHLGEAGALAAGAFALYEK | H |
|  |  | 842.4691 | 2524.386 | 3 | 2524.39 | -0.0044 | 62 | 80 | 0 | K | QHLGEAGALAAGAFALYEK | H |
|  |  | 842.4692 | 2524.386 | 3 | 2524.39 | -0.004 | 62 | 80 | 0 | K | QHLGEAGALAAGAFALYEK | H |
|  |  | 1102.584 | 2203.152 | 2 | 2203.158 | -0.0056 | 62 | 80 | 0 | K | QHLGEAGALAAGAFALYEK | H |
|  |  | 735.3914 | 2203.152 | 3 | 2203.158 | -0.0058 | 62 | 80 | 0 | K | QHLGEAGALAAGAFALYEK | H |
|  |  | 1102.583 | 2203.151 | 2 | 2203.158 | -0.0068 | 62 | 80 | 0 | K | QHLGEAGALAAGAFALYEK | H |
|  |  | 1102.583 | 2203.151 | 2 | 2203.158 | -0.0068 | 62 | 80 | 0 | K | QHLGEAGALAAGAFALYEK | H |
|  |  | 627.8476 | 2507.361 | 4 | 2507.363 | -0.0021 | 62 | 80 | 0 | K | QHLGEAGALAAGAFALYEK | H |
|  |  | 780.16 | 3116.611 | 4 | 3116.614 | -0.003 | 95 | 118 | 0 | K | ITEEIAATAAVGAGGYAFHEHHEK | K |
| gi|77553487 | Nonspecific lipid-transfer protein 2 precursor, putative, expressed | 1218.682 | 2435.349 | 2 | 2435.359 | -0.0108 | 79 | 98 | 0 | K | NVAGSISGLNAGNAASIPSK | C |
|  |  | 756.3995 | 1510.785 | 2 | 1510.789 | -0.0049 | 59 | 70 | 0 | R | SLNSAATTTADR | R |
|  |  | 756.4002 | 1510.786 | 2 | 1510.789 | -0.0035 | 59 | 70 | 0 | R | SLNSAATTTADR | R |
|  |  | 756.4001 | 1510.786 | 2 | 1510.789 | -0.0038 | 59 | 70 | 0 | R | SLNSAATTTADR | R |
|  |  | 812.7918 | 2435.354 | 3 | 2435.359 | -0.0057 | 79 | 98 | 0 | K | NVAGSISGLNAGNAASIPSK | C |
|  |  | 812.7918 | 2435.354 | 3 | 2435.359 | -0.0057 | 79 | 98 | 0 | K | NVAGSISGLNAGNAASIPSK | C |
|  |  | 812.7919 | 2435.354 | 3 | 2435.359 | -0.0054 | 79 | 98 | 0 | K | NVAGSISGLNAGNAASIPSK | C |
|  |  | 756.3995 | 1510.785 | 2 | 1510.789 | -0.0049 | 59 | 70 | 0 | R | SLNSAATTTADR | R |
|  |  | 1218.683 | 2435.35 | 2 | 2435.359 | -0.0089 | 79 | 98 | 0 | K | NVAGSISGLNAGNAASIPSK | C |
|  |  | 812.7917 | 2435.353 | 3 | 2435.359 | -0.0061 | 79 | 98 | 0 | K | NVAGSISGLNAGNAASIPSK | C |
| gi|19571117 | OSJNBb0008G24.11 | 741.8949 | 1481.775 | 2 | 1481.778 | -0.0029 | 54 | 65 | 0 | R | SEGAVAGAVDFR | G |
|  |  | 741.8953 | 1481.776 | 2 | 1481.778 | -0.0021 | 54 | 65 | 0 | R | SEGAVAGAVDFR | G |
|  |  | 741.8941 | 1481.774 | 2 | 1481.778 | -0.0045 | 54 | 65 | 0 | R | SEGAVAGAVDFR | G |
|  |  | 741.8939 | 1481.773 | 2 | 1481.778 | -0.0048 | 54 | 65 | 0 | R | SEGAVAGAVDFR | G |
| gi|77548426 | Nonspecific lipid-transfer protein precursor, putative, expressed | 889.0422 | 1776.07 | 2 | 1776.072 | -0.0021 | 87 | 99 | 0 | K | SLNLGAAAGIPGK | C |
|  |  | 889.0416 | 1776.069 | 2 | 1776.072 | -0.0032 | 87 | 99 | 0 | K | SLNLGAAAGIPGK | C |
| gi|18461235 | putative nuclear RNA binding protein A | 560.9916 | 1679.953 | 3 | 1679.956 | -0.0026 | 205 | 214 | 0 | K | VEESAPIAEK | Q |
|  |  | 739.4511 | 1476.888 | 2 | 1476.887 | 0.0003 | 37 | 47 | 0 | K | AAAAAAAPAGK | A |
|  |  | 840.983 | 1679.952 | 2 | 1679.956 | -0.004 | 205 | 214 | 0 | K | VEESAPIAEK | Q |
|  |  | 840.9839 | 1679.953 | 2 | 1679.956 | -0.0023 | 205 | 214 | 0 | K | VEESAPIAEK | Q |
|  |  | 424.283 | 1269.827 | 3 | 1269.827 | 0.0001 | 260 | 265 | 0 | K | ALLAFK | A |
|  |  | 840.9836 | 1679.953 | 2 | 1679.956 | -0.0029 | 205 | 214 | 0 | K | VEESAPIAEK | Q |
|  |  | 560.9918 | 1679.954 | 3 | 1679.956 | -0.0019 | 205 | 214 | 0 | K | VEESAPIAEK | Q |
| gi|70663913 | OSJNBa0029H02.25 | 735.8706 | 1469.727 | 2 | 1469.731 | -0.0038 | 621 | 630 | 0 | K | VVYDDGDVER | L |
|  |  | 817.9548 | 1633.895 | 2 | 1633.898 | -0.0031 | 34 | 44 | 0 | R | LIEEAETWLAR | V |
|  |  | 817.9543 | 1633.894 | 2 | 1633.898 | -0.0042 | 34 | 44 | 0 | R | LIEEAETWLAR | V |
| gi|125602537 | hypothetical protein OsJ_26407 | 1098.524 | 2195.034 | 2 | 2195.04 | -0.006 | 43 | 61 | 0 | K | DGPQEQYPAGAGAGETTSR | G |
|  |  | 732.6857 | 2195.035 | 3 | 2195.04 | -0.0044 | 43 | 61 | 0 | K | DGPQEQYPAGAGAGETTSR | G |
|  |  | 655.373 | 1963.097 | 3 | 1963.099 | -0.0017 | 228 | 238 | 0 | K | QDPQFLLEHTK | K |

^a^Exp_mz indicates mass-to-charge ratio measured in the experiment. ^b^Exp_mr indicates mass of the peptide measured in the experiment. ^c^Z indicates charge of the peptide. ^d^Calc_mr indicates mass of the peptide calculated theoretically. ^e^Delta indicates deviation between masses of the peptide measured in the experiment and calculated theoretically. ^f^Start indicates the starting location of the peptide in the protein sequence. ^g^End indicates the ending location of the peptide in the protein sequence. ^h^Miss indicates the number of the missing peptide during the digestion.

^i^Before indicates the first amino acid before the peptide. ^j^Seq indicates the sequence of the peptide. ^k^After indicates the first amino acid after the peptide.
