# Supplementary material for: Quantitative Proteomic Analysis of the Rice (Oryza sativa L.) Salt Response
Source: PLoS One. 2015 Mar 20;10(3):e0120978. doi: 10.1371/journal.pone.0120978 (PMC4368772; doi:10.1371/journal.pone.0120978)
Supplement: S6 Table — (DOC) [file pone.0120978.s006.doc]

**S6 Table. The homologue of the differentially expressed proteins.**

| Accession | Description | Query Accession | Query Definition | Cov^a^ | Ident^b^ |
| --- | --- | --- | --- | --- | --- |
| gi|29367391 | chloroplast photosystem I reaction center subunit II precursor-like protein (PsaD) | gi|410935703 | photosystem I reaction center subunit II [Saccharum hybrid cultivar GT28] | 100% | 86% |
|  |  | gi\|15218708 | photosystem I reaction center subunit D-2 [Arabidopsis thaliana] | 99% | 72% |
| gi|3885894 | photosystem-1 H subunit GOS5 (PsaH) | gi|475583817 | Photosystem I reaction center subunit VI, chloroplastic [Aegilops tauschii] | 100% | 86% |
|  |  | gi\|15218186 | photosystem I reaction center subunit H-2 [Arabidopsis thaliana] | 100% | 71% |
| gi|3789954 | chlorophyll a/b-binding protein precursor (Lhca1) | gi|226503327 | chlorophyll a-b binding protein 6A [Zea mays] | 99% | 93% |
|  |  | gi\|15233115 | chlorophyll a-b binding protein 6 [Arabidopsis thaliana] | 84% | 83% |
| gi|34393511 | putative photosystem I antenna protein (Lhca2) | gi|2306981 | photosystem I antenna protein [Oryza sativa Indica Group] | 100% | 91% |
|  |  | gi\|6850838 | Lhca2 protein [Arabidopsis thaliana] | 84% | 89% |
| gi|3789952 | chlorophyll a/b-binding protein precursor (Lhca4) | gi|157361232 | photosystem I light-harvesting complex type 4 protein [Bambusa oldhamii] | 100% | 87% |
|  |  | gi\|30692874 | chlorophyll a-b binding protein 4 [Arabidopsis thaliana] | 99% | 73% |
| gi|18855008 | putative chloroplast chaperonin | gi|195621498 | CHL-CPN10 [Zea mays] | 100% | 84% |
|  |  | gi\|18406593 | chloroplast chaperonin 10 [Arabidopsis thaliana] | 100% | 54% |
| gi|51090743 | putative peroxiredoxin Q | gi|475605309 | Peroxiredoxin Q, chloroplastic [Aegilops tauschii] | 100% | 92% |
|  |  | gi\|15230982 | peroxiredoxin Q [Arabidopsis thaliana] | 70% | 82% |
| gi|46389828 | putative thioredoxin peroxidase | gi|473825951 | Peroxiredoxin-2E-1, chloroplastic [Triticum urartu] | 63% | 86% |
|  |  | gi\|15231718 | peroxiredoxin-2E [Arabidopsis thaliana] | 95% | 65% |
| gi|57899183 | thioredoxin M-like | gi|6143893 | thioredoxin-like protein [Arabidopsis thaliana] | 88% | 76% |
|  |  | gi\|42563272 | thioredoxin Y1 [Arabidopsis thaliana] | 92% | 74% |
| gi|32487506 | thioredoxin x | gi|195624046 | thioredoxin X [Zea mays] | 100% | 74% |
|  |  | gi\|18403021 | thioredoxin X [Arabidopsis thaliana] | 69% | 63% |
| gi|11177845 | putative glutathione S-transferase OsGSTF3 | gi|162463945 | glutathione transferase10 [Zea mays] | 75% | 89% |
| gi|3885882 | inorganic pyrophosphatase | gi|388271212 | inorganic pyrophosphotase [Triticum aestivum] | 100% | 93% |
| gi|28564802 | putative vacuolar ATP synthase subunit H | gi|110559322 | vacuolar proton ATPase subunit H [Triticum aestivum] | 99% | 92% |
|  |  | gi\|15228443 | V-type proton ATPase subunit H [Arabidopsis thaliana] | 100% | 71% |
| gi|51535416 | putative ATP synthase delta chain | gi|475609344 | ATP synthase subunit O, mitochondrial [Aegilops tauschii] | 100% | 76% |
|  |  | gi\|15240628 | delta subunit of Mt ATP synthase [Arabidopsis thaliana] | 89% | 55% |
| gi|41052565 | putative ATP synthase | gi|47607439 | mitochondrial ATP synthase precursor [Triticum aestivum] | 100% | 86% |
|  |  | gi\|15227104 | mitochondrial F1F0-ATP synthase subunit Fad [Arabidopsis thaliana] | 100% | 66% |
| gi|108864431 | Acyl carrier protein 2, chloroplast precursor | gi|226494889 | acyl carrier protein 2 [Zea mays] | 91% | 71% |
|  |  | gi\|15234875 | acyl carrier protein 4 [Arabidopsis thaliana] | 78% | 59% |
| gi|77553225 | Carboxyvinyl-carboxyphosphonate phosphorylmutase, putative, expressed | gi|590599983 | Phosphoenolpyruvate carboxylase family protein [Theobroma cacao] | 79% | 72% |
|  |  | gi\|18411340 | carboxyvinyl-carboxyphosphonate phosphorylmutas [Arabidopsis thaliana] | 79% | 71% |
| gi|24431603 | Putative transcription factor | gi|409194148 | basic transcription factor 3 [Triticum aestivum] | 24% | 94% |
| gi|55296302 | putative MAR binding filament-like protein 1 | gi|350537767 | MAR-binding filament-like protein 1 [Solanum lycopersicum] | 86% | 36% |
|  |  | gi\|42564228 | MAR-binding filament-like protein 1 [Arabidopsis thaliana] | 93% | 35% |
| gi|77552436 | auxin-repressed protein-like protein ARP1, putative, expressed | gi|541905589 | auxin repressed protein [Oryza sativa Indica Group] | 100% | 97% |
|  |  | gi\|18396748 | dormancy-associated protein-like 1 [Arabidopsis thaliana] | 100% | 55% |
| gi|33358444 | hydroperoxide lyase | gi|162462890 | hydroperoxide lyase1 [Zea mays] | 100% | 73% |
|  |  | gi3822403 | hydroperoxide lyase [Arabidopsis thaliana] | 95% | 53% |
| gi|34015153 | putative CBS domain containing protein | gi|703143017 | CBS domain-containing protein CBSX1 [Morus notabilis] | 85% | 80% |
|  |  | gi\|442570759 | Chain A, Crystal Structure Of Cbs-pair Protein, Cbsx1 [Arabidopsis thaliana] | 83% | 81% |
| gi|28209481 | expressed protein | gi|195627228 | EF hand family protein [Zea mays] | 93% | 90% |
|  |  | gi\|18406507 | calcium-binding EF-hand-containing protein [Arabidopsis thaliana] | 87% | 55% |
| gi|5922611 | putative small GTP-binding protein Bsar1a | gi|187424044 | GTPase SAR1 [Triticum aestivum] | 100% | 95% |
|  |  | gi\|15235226 | GTP-binding protein SAR1A [Arabidopsis thaliana] | 100% | 93% |
| gi|50878396 | putative P-II nitrogen sensing protein | gi|13277515 | PII protein [Medicago sativa] | 60% | 84% |
|  |  | gi\|134105056 | Chain A, Crystal Structure Of Pii Bound To Citrate [Arabidopsis thaliana] | 60% | 75% |
| gi|41052905 | putative small nuclear ribonucleoprotein polypeptide D3 | gi|673921573 | small nuclear ribonucleoprotein Sm D3-like [Zea mays] | 100% | 98% |
|  |  | gi\|15223010 | snRNP core protein SMD3 [Arabidopsis thaliana] | 100% | 72% |
| gi|113578236 | Os05g0154800 | gi|475574987 | U1 small nuclear ribonucleoprotein A [Aegilops tauschii] | 92% | 86% |
|  |  | gi\|15226631 | spliceosomal protein U1A [Arabidopsis thaliana] | 98% | 70% |
| gi|50725625 | putative acidic ribosomal protein P1a | gi|162461632 | 60S acidic ribosomal protein P1 [Zea mays] | 100% | 83% |
|  |  | gi\|18411448 | 60S acidic ribosomal protein P1-2 [Arabidopsis thaliana] | 96% | 58% |
| gi|25553579 | putative ribosomal protein S18 | gi|226504536 | 40S ribosomal protein S18 [Zea mays] | 100% | 99% |
|  |  | gi\|15219950 | 40S ribosomal protein S18 [Arabidopsis thaliana] | 100% | 87% |
| gi|50252685 | putative ribosomal protein L10a | gi|474392083 | 60S ribosomal protein L10a-1 [Triticum urartu] | 100% | 93% |
|  |  | gi\|30680605 | 60S ribosomal protein L10a-1 [Arabidopsis thaliana] | 100% | 88% |
| gi|11974 | ribosomal protein S2 | gi|556927078 | 30S ribosomal protein S2 [Oryza rufipogon] | 100% | 100% |
| gi|37805854 | putative ribosomal protein L34 | gi|226499450 | 60S ribosomal protein L34 [Zea mays] | 100% | 97% |
| gi|14495192 | putative 26S proteasome subunit RPN9b | gi|17298163 | 26S proteasome regulatory particle non-ATPase subunit9b [Oryza sativa Japonica Group] | 72% | 96% |
|  |  | gi\|30684612 | proteasome component (PCI) domain-containing protein [Arabidopsis thaliana] | 99% | 76% |
| gi|11094192 | 26S proteasome regulatory particle triple-A ATPase subunit4 | gi|226505536 | 26S protease regulatory subunit S10B [Zea mays] | 100% | 98% |
| gi|12039318 | histone H4 | gi|195617694 | histone H4 [Zea mays] | 100% | 100% |
|  |  | gi\|15226944 | histone H4 [Arabidopsis thaliana] | 100% | 100% |
| gi|6319146 | H2A protein | gi|195607344 | histone H2A variant 3 [Zea mays] | 100% | 87% |
|  |  | gi\|15232536 | histone H2A 11 [Arabidopsis thaliana] | 100% | 86% |
| gi|3885890 | histone H3 | gi|413937382 | histone H3 [Zea mays] | 100% | 100% |
|  |  | gi\|15236103 | Histone H3.3 [Arabidopsis thaliana] | 100% | 100% |
| gi|29124123 | putative actin depolymerizing factor | gi|226500484 | actin-depolymerizing factor 3 [Zea mays] | 100% | 75% |
|  |  | gi\|30697300 | actin depolymerizing factor 4 [Arabidopsis thaliana] | 99% | 62% |
| gi|34851127 | actin | gi|474259583 | Actin-3 [Triticum urartu] | 100% | 99% |
|  |  | gi\|15242516 | actin 7 [Arabidopsis thaliana] | 100% | 98% |
| gi|27260946 | putative isopentenyl pyrophosphate: dimethyllallyl pyrophosphate isomerase | gi|414887012 | isopentenyl pyrophosphate isomeraseIsopentenyl-diphosphate delta-isomerase II [Zea mays] | 91% | 95% |
|  |  | gi\|15232981 | Isopentenyl-diphosphate Delta-isomerase II [Arabidopsis thaliana] | 98% | 88% |
| gi|34393921 | putative isocitrate lyase | gi|283806369 | isocitrate lyase [Triticum aestivum] | 100% | 91% |
|  |  | gi\|30686361 | isocitrate lyase [Arabidopsis thaliana] | 100% | 78% |
| gi|5257275 | putative caffeoyl-CoA O-methyltransferase 1 | gi|565410650 | caffeoyl CoA O-methyltransferase 2 [Panicum virgatum] | 96% | 80% |
|  |  | gi\|21595512 | caffeoyl-CoA O-methyltransferase-like protein [Arabidopsis thaliana] | 91% | 58% |
| gi|21686526 | ferritin | gi|210061129 | ferritin 1A [Triticum aestivum] | 78% | 93% |
| gi|125600465 | hypothetical protein OsJ_24479 | gi|573950962 | PREDICTED: protein LHCP TRANSLOCATION DEFECT-like [Oryza brachyantha] | 99% | 89% |
|  |  | gi\|18403267 | protein GRANA DEFICIENT CHLOROPLAST 1 [Arabidopsis thaliana] | 79% | 68% |
| gi|62701927 | CBS domain, putative | gi|226501428 | CBS domain containing protein [Zea mays] | 90% | 72% |
|  |  | gi\|15227986 | CBS / octicosapeptide/Phox/Bemp1 domain-containing protein [Arabidopsis thaliana] | 81% | 62% |
| gi|50252988 | unknown protein | gi|413923438 | hypothetical protein ZEAMMB73_554585 [Zea mays] | 77% | 81% |
|  |  | gi\|15241700 | uncharacterized protein [Arabidopsis thaliana] | 71% | 55% |
| gi|125590644 | hypothetical protein OsJ_15076 | gi|162462211 | prohibitin2 [Zea mays] | 100% | 95% |
|  |  | gi\|15237488 | prohibitin2 [Arabidopsis thaliana] | 97% | 78% |
| gi|53749372 | unknown protein | gi|413952234 | USP family protein [Zea mays] | 100% | 76% |
|  |  | gi\|30682187 | adenine nucleotide alpha hydrolases-like protein [Arabidopsis thaliana] | 93% | 55% |
| gi|56784479 | hypothetical protein | gi|590591783 | NAD(P)H-quinone oxidoreductase subunit N [Theobroma cacao] | 34% | 80% |
|  |  | gi\|15237153 | NADH dehydrogenase-like complex N [Arabidopsis thaliana] | 43% | 65% |
| gi|62701926 | abscisic acid- and stress-induced protein | gi|149391461 | abscisic stress ripening protein 2 [Oryza sativa Indica Group] | 80% | 100% |
| gi|77553487 | Nonspecific lipid-transfer protein 2 precursor, putative, expressed | gi|2407273 | lipid transfer protein LPT II [Oryza sativa Indica Group] | 100% | 97% |
| gi|19571117 | OSJNBb0008G24.11 | gi|414879481 | TPA: hypothetical protein ZEAMMB73_385814 [Zea mays] | 91% | 82% |
| gi|77548426 | Nonspecific lipid-transfer protein precursor, putative, expressed | gi|47155247 | lipid transfer protein [Oryza sativa Indica Group] | 100% | 92% |
| gi|18461235 | putative nuclear RNA binding protein A | gi|659928057 | plasminogen activator inhibitor 1 RNA-binding protein [Zea mays] | 99% | 76% |
| gi|70663913 | OSJNBa0029H02.25 | gi|116310758 | H0311C03.6 [Oryza sativa Indica Group] | 100% | 99% |
| gi|125602537 | hypothetical protein OsJ_26407 | gi|218200659 | hypothetical protein OsI_28219 [Oryza sativa Indica Group] | 100% | 99% |
|  |  | gi\|240255391 | uncharacterized protein [Arabidopsis thaliana] | 67% | 56% |

The differentially expressed proteins were searched in NCBI (http://www.ncbi.nlm.nih.gov/) with blastp.

^a^Cov indicates percentage of query protein to the predicted protein.

^b^Ident indicates percentage of matched sequence to the predicted protein.
