# Supplementary figures and images for: Quantitative Proteomic Analysis of the Rice (Oryza sativa L.) Salt Response
Source: PLoS One. 2015 Mar 20;10(3):e0120978. doi: 10.1371/journal.pone.0120978 (PMC4368772; doi:10.1371/journal.pone.0120978)

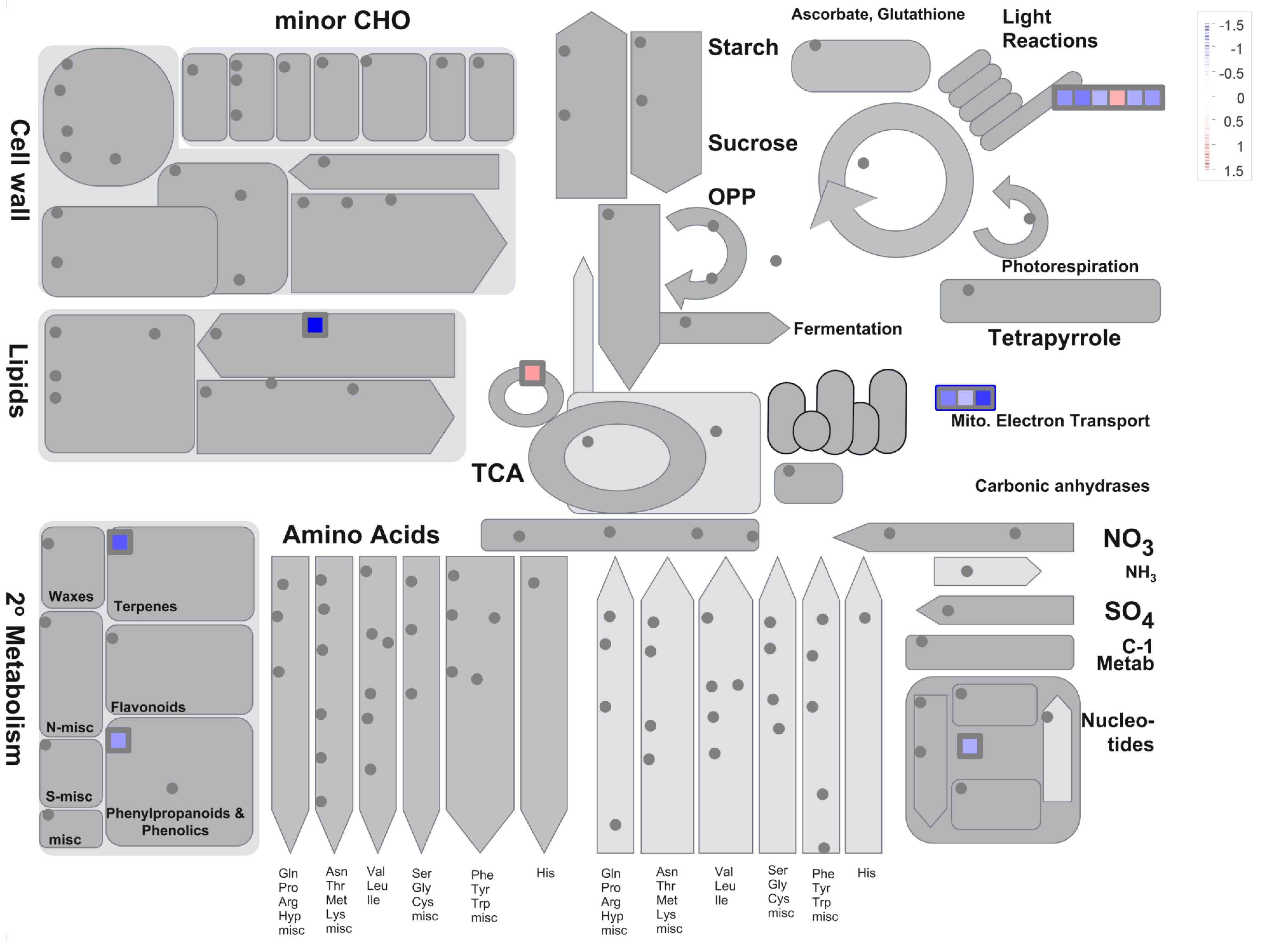

Supplement: S1 Fig — (TIF) [file pone.0120978.s007.tif]

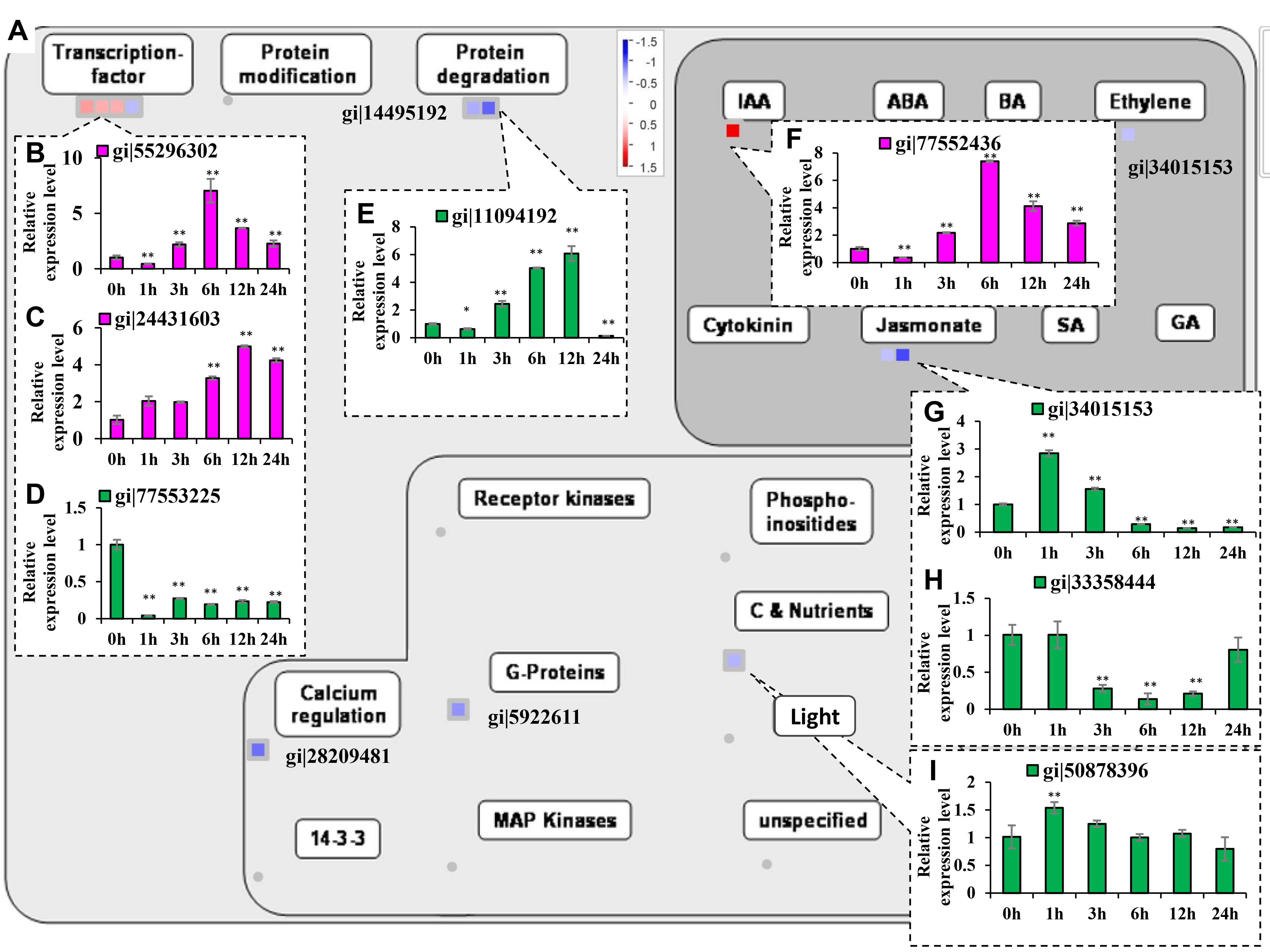

Supplement: S2 Fig — The transcript abundances of mRNAs encoding proteins involved in regulation were analyzed at 0 h, 1 h, 3 h, 6 h, 12 h and 24 h following salt stress treatment. The mRNA levels at 12 h were compared with the iTRAQ data. Red indicates the proteins that were up-regulated and green indicates the proteins that were down-regulated. Significant differences were determined relative to each treatment using a student’s t-test [P-values <0.05 (*) and <0.01 (**)]. Bars: SD. The changes in transcript abundances at 12 h were compared with the iTRAQ data. (A) Overview of differentially expressed proteins involved in regulation. (B) Putative MAR binding filament-like protein 1 (gi|55296302). (C) Putative transcription factor (gi|24431603). (D) Carboxyvinyl-carboxyphosphonate phosphorylmutase (gi|77553225). (E) 26S proteasome regulatory particle triple-A ATPase subunit 4 (gi|11094192). (F) Auxin-repressed protein-like protein ARP1 (gi|77552436). (G) Putative CBS domain containing protein (gi|34015153). (H) Hydroperoxide lyase (gi|33358444). (I) Putative P-II nitrogen sensing protein (gi|50878396). (TIF) [file pone.0120978.s008.tif]

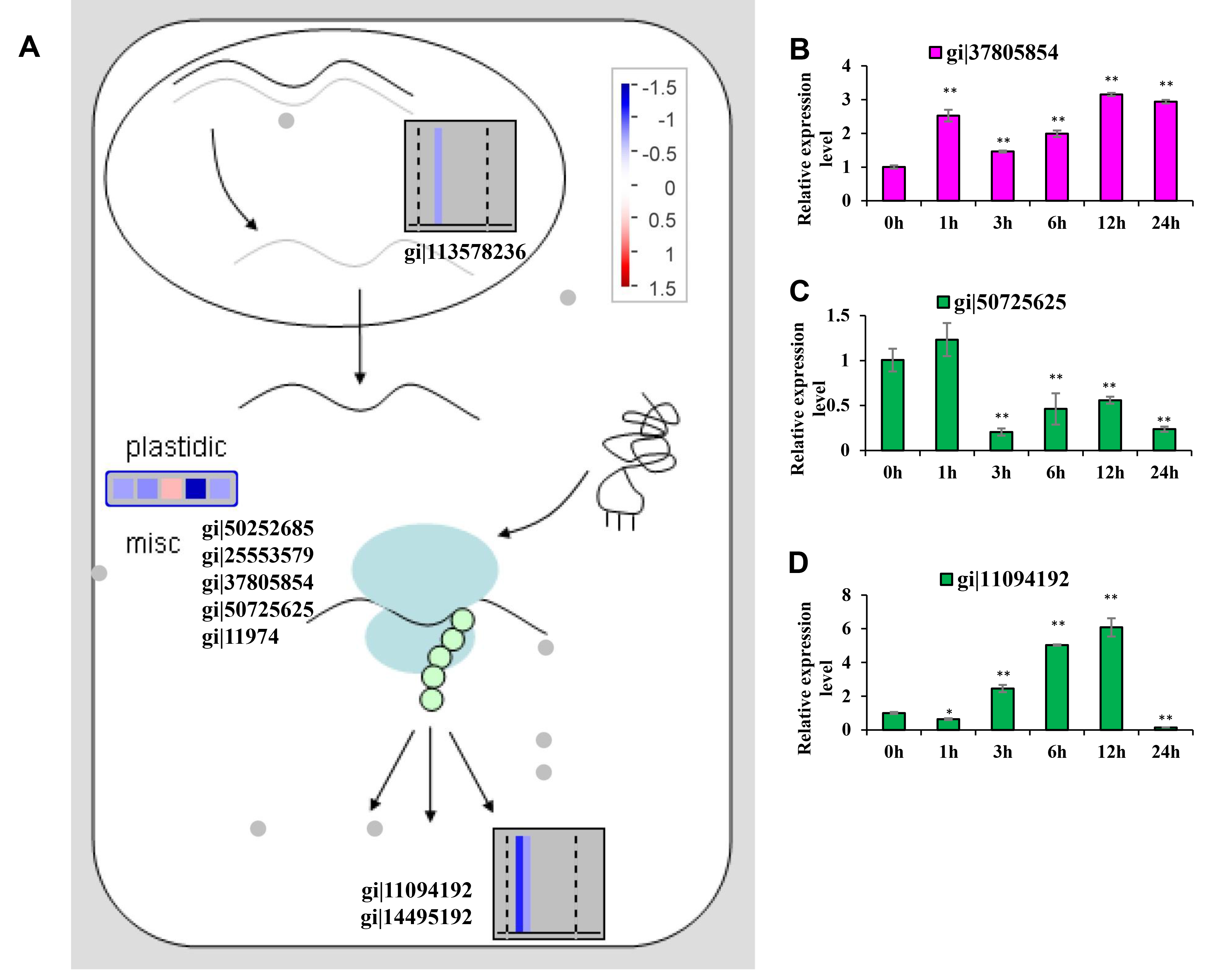

Supplement: S3 Fig — The transcript abundances of mRNAs encoding proteins involved in RNA and protein synthesis were analyzed at 0 h, 1 h, 3 h, 6 h, 12 h and 24 h following salt stress treatment. The mRNA levels at 12 h were compared with the iTRAQ data. Red indicates the proteins that were up-regulated and green indicates the proteins that were down-regulated. Significant differences were determined relative to each treatment using a student’s t-test [P-values <0.05 (*) and <0.01 (**)]. Bars: SD. The changes in transcript abundances at 12 h were compared with the iTRAQ data. (A) Overview of differentially expressed proteins involved in RNA and protein synthesis. (B) Putative ribosomal protein L34 (gi|37805854). (C) Putative acidic ribosomal protein P1a (gi|50725625). (D) 26S proteasome regulatory particle triple-A ATPase subunit 4 (gi|11094192). (TIF) [file pone.0120978.s009.tif]

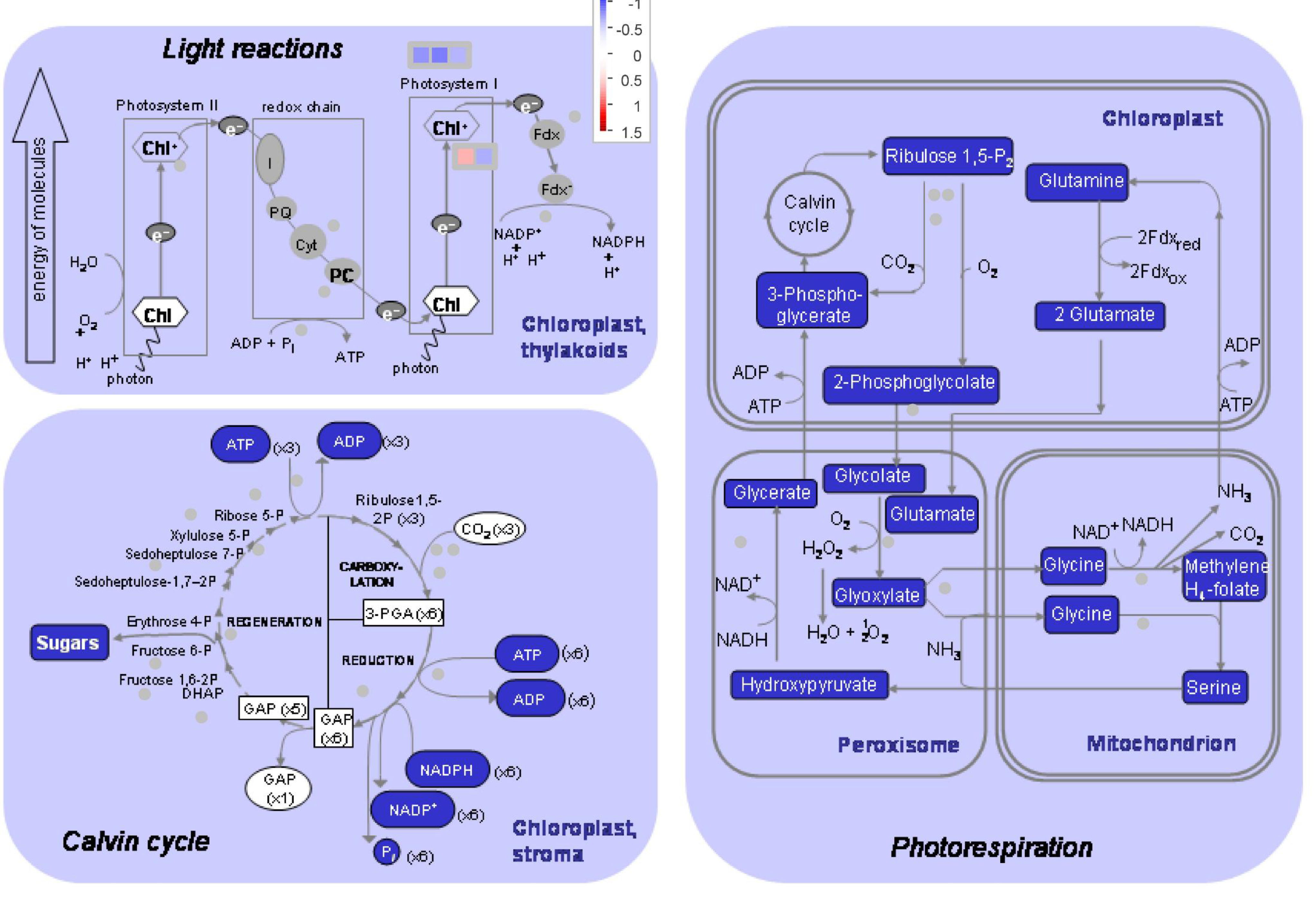

Supplement: S4 Fig — (TIF) [file pone.0120978.s010.tif]

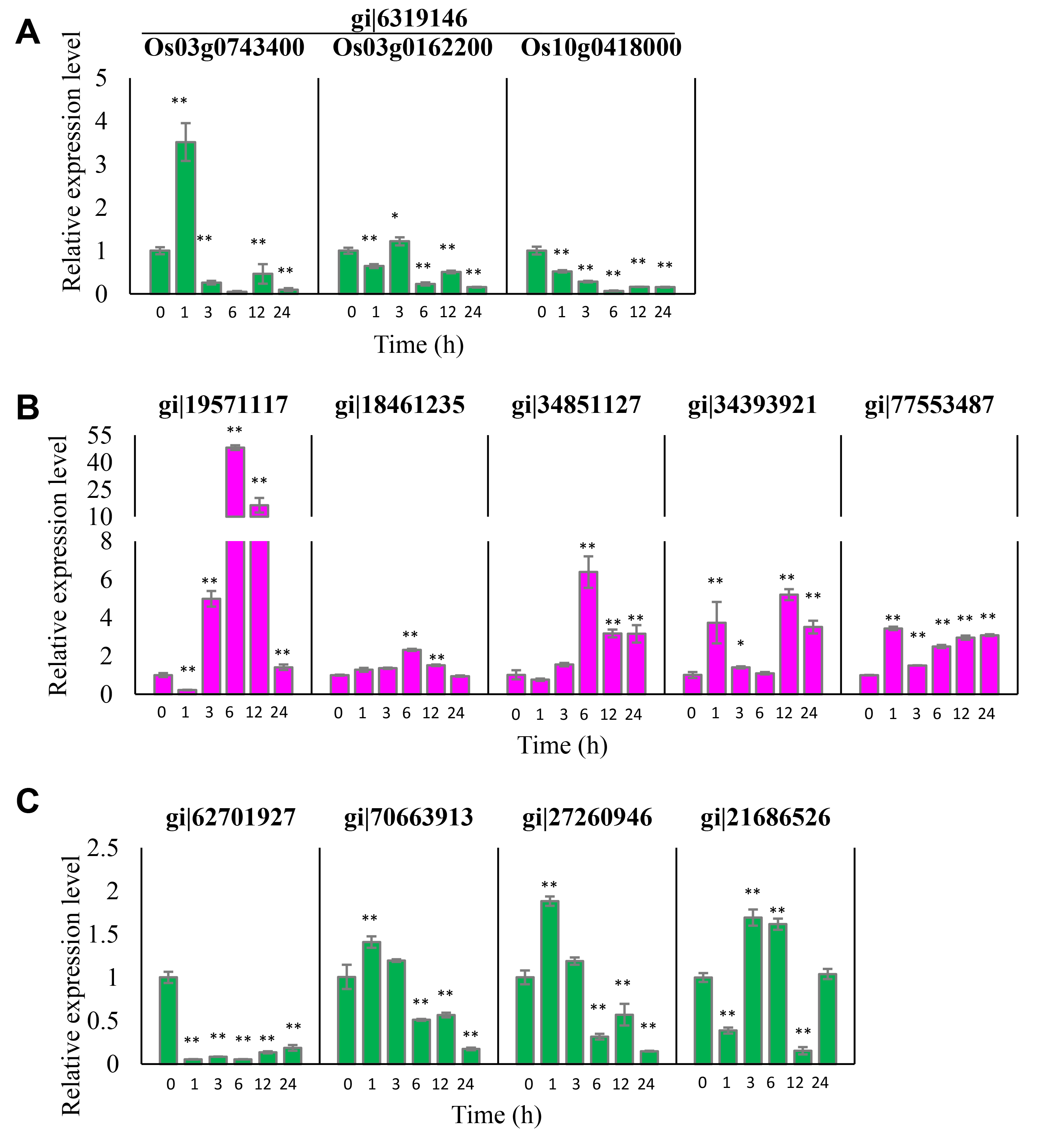

Supplement: S5 Fig — The transcript abundances of mRNAs encoding the differentially expressed proteins were analyzed at 0 h, 1 h, 3 h, 6 h, 12 h and 24 h following salt stress treatment. The mRNA levels at 12 h were compared with the iTRAQ data. Red indicates the proteins that were up-regulated and green indicates the proteins that were down-regulated. Significant differences were determined relative to each treatment using a student’s t-test [P-values <0.05 (*) and <0.01 (**)]. Bars: SD. The changes in transcript abundances at 12 h were compared with the iTRAQ data. (A) qRT-PCR analysis of mRNA encoding H2A protein (gi|6319146). (B) qRT-PCR analysis of mRNAs encoding up-regulated proteins OSJNBb0008G24.11 (gi|19571117), putative nuclear RNA binding protein A (gi|18461235), Actin (gi|34851127), putative Isocitrate lyase (gi|34393921) and nonspecific lipid-transfer protein 2 precursor (gi|77553487). (C) qRT-PCR analysis of mRNAs encoding down-regulated proteins CBS domain (gi|62701927), OSJNBa0029H02.25 (gi|70663913), putative isopentenyl pyrophosphate: dimethyllallyl pyrophosphate isomerase (gi|27260946) and ferritin (gi|21686526). (TIF) [file pone.0120978.s011.tif]
